# AL amyloidosis clonal plasma cells are regulated by microRNAs and dependent on anti‐apoptotic BCL2 family members

**DOI:** 10.1002/cam4.5621

**Published:** 2023-01-24

**Authors:** Hila Fishov, Eli Muchtar, Mali Salmon‐Divon, Angela Dispenzieri, Tal Zvida, Claudio Schneider, Benjamin Bender, Adrian Duek, Merav Leiba, Ofer Shpilberg, Oshrat Hershkovitz‐Rokah

**Affiliations:** ^1^ Department of Molecular Biology, Faculty of Natural Sciences Ariel University Ariel Israel; ^2^ Translational Research Lab, Assuta Medical Centers Tel‐Aviv Israel; ^3^ Division of Hematology Department of Internal Medicine, Mayo Clinic Rochester Minnesota USA; ^4^ Adelson School of Medicine Ariel University Ariel Israel; ^5^ Orthopedic Department, Assuta Medical Centers Tel‐Aviv Israel; ^6^ Institute of Hematology Assuta Ashdod University Hospital, Faculty of Health Science Ben‐Gurion University of the Negev Beer Sheva Israel; ^7^ Institute of Hematology, Assuta Medical Centers Tel‐Aviv Israel

**Keywords:** AL amyloidosis, BCL2 family, multiple myeloma, noncoding RNA

## Abstract

**Background:**

Noncoding RNAs such as microRNAs (miRNAs) have attracted attention as biological pathway regulators, which differ from chromosomal translocations and gene point mutations. Their involvement in the molecular mechanisms underlying light chain (AL) amyloidosis pathogenesis is yet to be elucidated.

**Aims:**

To decipher specific miRNA expression profile in AL‐amyloidosis and to examine how miRNAs are involved in AL pathogenesis.

**Methods:**

The expression profile of miRNAs and mRNA from bone marrow (BM)‐derived CD138+ cells were determined using the NanoString nCounter assay and RNA‐Seq, respectively. The effect of aberrantly expressed miRNAs on potential molecular targets was analyzed by qRT‐PCR, Western blot, Mito‐potential assay, and Annexin‐PI staining.

**Results:**

Genes which were significantly differentially expressed between AL‐amyloidosis and MM, were found to be involved in cell growth and apoptotic mechanisms. Specifically, BCL2L1, MCL1, and BCL2 were upregulated in AL‐amyloidosis compared with MM and controls. The levels of miR‐181a‐5p and miR‐9‐5p, which regulate the above‐mentioned genes, were lower in BM samples from AL‐amyloidosis compared with controls, providing a mechanism for BCL2 family gene upregulation. When miR‐9‐5p and miR‐181a‐5p were overexpressed in ALMC1 cells, BCL2L1, MCL1, and BCL2 were downregulated and induced apoptosis. Treatment of ALMC‐1 cells with venetoclax, (BCL‐2 inhibitor), resulted in the upregulation of those miRNAs, the downregulation of BCL2, MCL1, and BCL2L1 mRNA and protein levels, and subsequent apoptosis.

**Conclusion:**

Our findings suggest that miR‐9‐5p and miR‐181a‐5p act as tumor‐suppressors whose downregulation induces anti‐apoptotic mechanisms underlying the pathogenesis of AL‐amyloidosis. The study highlights the post‐transcriptional regulation in AL‐amyloidosis and provides pathogenetic evidence for the potential use of BCL‐2 inhibitors in this disease.

## Graphical abstract

BCL2 family members are highly expressed in AL amyloidosis compared with MM, underpinning the therapeutic potential of BCL2 inhibitors in this disease. miR‐9‐5p and miR‐181a‐5p act as tumor suppressors in AL amyloidosis by inducing apoptotic mechanisms.

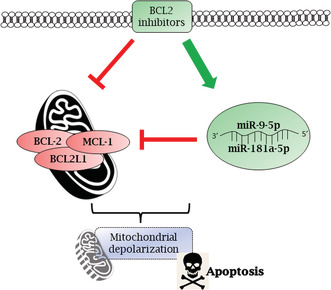

## 1 INTRODUCTION

Amyloidosis is a disorder characterized by extracellular accumulation of aggregated and misfolded autologous proteins in organs, eventually leading to their dysfunction.^1^ There are more than 30 types of systemic amyloidosis. Immunoglobulin light chain (AL) amyloidosis is a common and severe type of systemic amyloidosis caused by circulating free immunoglobulin light chains derived from bone marrow (BM) clonal plasma cells. AL amyloidosis is associated with substantial morbidity and mortality,^2^ and patients are often diagnosed late in their disease course, where therapies are unlikely to reverse organ damage and thus offer little benefit. AL amyloidosis is closely related to multiple myeloma (MM); the overlap between the two disorders is seen in up to 17% of patients.^3^ There is little known on AL pathogenesis from the underlying plasma cell disorder perspective, and the similarities and differences from MM.

Current treatments of AL amyloidosis have mostly been adapted from treatment regimens developed and used in MM.^4^, ^5^ However, these do not target the already deposited amyloid. Hence, there is a substantial unmet need for novel treatments for these patients.

MicroRNAs (miRNAs) are short noncoding RNA molecules (~22 nucleotides) that regulate gene expression post‐transcriptionally by inducing mRNA cleavage and degradation.^6^ miRNAs are essential components in pathways involved in cancer initiation, progression, and treatment response or resistance.^7^, ^8^, ^9^ Some of the inherent properties of miRNAs render them greatly attractive as potential biomarkers: they can be isolated from various bio‐fluids, are highly stable in cell‐free form, and can be easily identified in small‐volume samples.^7^ Moreover, the understanding of the interactions between miRNA and mRNA may contribute to elucidate the molecular mechanism involved in disease pathogenesis and represent a potential new therapeutic approach. Many studies have shown that the dysregulation of miRNAs has an important role in tumorigenesis of hematological malignancies^10^ including MM.^11^ However, the role of miRNA dysregulation in AL amyloidosis has been little studied.^12^, ^13^ Here, we investigated the expression profile of miRNAs and mRNA from BM samples of individuals with AL amyloidosis and MM.

## 2 MATERIALS AND METHODS

### 2.1 Samples

Bone marrow samples were collected from 73 newly diagnosed AL amyloidosis patients and from 80 newly diagnosed MM patients. The samples were collected at Mayo Clinic (Rochester, MN, USA) and Assuta Medical Centers (Tel‐Aviv and Ashdod, Israel) in accordance with the local Institutional Review Board protocols. BM from healthy controls (HCs) was obtained from individuals undergoing hip replacement as previously described.^14^ All patients provided informed consent for their sample collection and medical chart review for research purposes.

### 2.2 Isolation of CD138+ cells, RNA extraction, and quantitative polymerase chain reaction (RT‐PCR)

Ammonium‐Chloride‐Potassium (ACK) buffer was used for the lysis of red blood cells in BM samples. Then, CD138+ cells were isolated using RoboSep EasySep ® magnetic Nanoparticles [(#17877RF) Stemcell Technologies], and sorted by RoboSep‐S™ cell separation system (Stemcell Technologies) according to the manufacturer's protocol. The average purity of the plasma cells was 79% with only minority of the samples (8%) below 50% purity. To maintain RNA integrity the sorted CD138+ cells were frozen in RNA‐Later solution (Thermo Fisher Scientific Inc.). RNA was purified using RNAeasy purification kit (Qiagen). RNA concentrations were measured by Qubit spectrophotometer (Thermo Fisher Scientific Inc.). MiRNAs and mRNA detection by qRT‐PCR was performed as described in the Appendix S1.

### 2.3 Cell lines

AL amyloidosis cell line, ALMC‐1, was kindly provided by Prof. Diane Jelinek (Mayo Clinic, Phoenix, AZ, USA). The cells were cultured in Iscove's Modified Dulbecco's Medium (Gibco, Thermo Fisher Scientific Inc.) supplemented with 10% fetal bovine serum (FBS; Biological Industries), 2 mM glutamine, 1% penicillin and streptomycin and 1 ng/ml human interleukin‐6 (PeproTech). The cells were cultured at 37°C in a humidified incubator with 5% CO_2._

### 2.4 NanoString analysis

The multiplexed nCounter® miRNA Expression Assay kit (NanoString Technologies) was used to profile 800 mature human miRNAs. Read counts from each sample were normalized to the geometric mean of the top 100 highest expressed miRNAs followed by voom transformation.^15^ Then, we used RUV2^16^ to detect unwanted variation using the positive spike‐in from Illumina and ACTB as housekeeping genes. The mean value of negative controls was calculated for each sample. Only miRNAs having expression values higher than the noise in all samples were kept for downstream analysis. Differentially expressed miRNAs were detected by applying linear models as implemented in limma R package,^17^ the estimated unwanted variation detected by RUV2 was included in the design matrix to remove the batch effect. MiRNAs having fold change of at least 2 and FDR <0.1 were considered as differentially expressed.

### 2.5 RNA sequencing

SMARTer Stranded Total RNA‐Seq Kit v2—Pico Input Mammalian (Takara‐Clontech) was used for the preparation of RNA sample libraries according to the manufacturer's protocol. The NexSeq platform (Illumina) was used for RNA sequencing (RNA‐seq). Quality control, read mapping, and differential expression analysis were performed as described before^18^ with the following changes; clean reads were mapped to the human genome (hg38) using HISAT2.^19^ The number of reads mapping each human gene (as annotated in the gencode v29 annotation) was counted with the feature Counts program.^20^ The count table was filtered to contain only mRNAs having at least one count per million (CPM) in at least four samples. Genes with an FDR <0.05 and a fold change>2 were considered as being differentially expressed.

For visualization of the subgroup classification, heatmaps were generated using heatmap.2 from gplots version 3.0.4 R package, by using unsupervised hierarchical clustering (ward.D2 linkage) and 1‐correlation as the distance method. The RNA‐Seq raw reads were uploaded to NCBI (SRA accession no. PRJNA846139).

### 2.6 Public dataset of gene expression

Publicly available count table of RNA‐seq data generated from plasma cells of 32 AL and 32 MM patients was downloaded from the Gene Expression Omnibus database (GSE175384). The count table was filtered to include only genes showing 1 CPM in at least 32 samples. Normalization and detection of differentially expressed genes between AL and MM were done similar to the RNA‐seq analysis described above.

### 2.7 Cell transfections with miRNA mimics

Cells were transfected with 50 nM miR‐9‐5p mimic, miR‐181a‐5p mimic, or scramble sequence for negative control (Applied Biosystems, Thermo Fisher Scientific Inc.) using lipofectamine 3000 (Invitrogen, Thermo Fisher Scientific Inc.) according to the manufacturer's protocol.

### 2.8 Compound and cell proliferation assay

Venetoclax (V‐3579, LC Laboratories) was dissolved in DMSO. Cell proliferation assay was performed as described in the Appendix S1. For downstream experiments, venetoclax was added to the cells at a final concentration of 0.5 and 1 μM.

### 2.9 Western blot analysis

Cells (2 × 10^5^) were harvested using SingleShot™ Cell Lysis Kit (Bio‐Rad). Western blot analysis was performed as described in the Appendix S1.

### 2.10 Apoptosis and mitochondrial membrane potential assay

For apoptosis assay, cells were transfected with the miRNA mimic or treated with venetoclax. After 48–72 h cells were harvested and washed with PBS. The assay was performed as described in the Appendix S1.

Changes in mitochondrial potential and cellular plasma membrane permeabilization were determined with the Muse® Mitochondrial Kit (Luminex). Briefly, ALMC‐1 cells were treated with venetoclax or transfected with the miR‐9‐5p mimic or miR‐181a‐5p mimic for 48 h. The cells were incubated with MitoPotential working solution for 20 min at 37°C in 5% CO_2_. Then, 5 μl Muse® MitoPotential 7‐ADD reagent was added and the cells were incubated for 5 min at room temperature. The percentage of four cell populations: live (mitopotential^+^/7‐AAD^−^), depolarized live (mitopotential^−^/7‐AAD^−^), dead (mitopotential^+^/7‐AAD^+^), and depolarized dead (mitopotential^−^/7‐AAD^+^) were measured using the Muse® Cell Analyzer (Luminex).

### 2.11 Bioinformatics analysis

MiRNAs targets and their biological relevance were identified using the bioinformatics tools: ingenuity pathway analysis (IPA),^21^ miRTar Base,^22^ and TargetScan.^23^

### 2.12 Statistical analysis

Data are presented as means ± standard error of the mean (SEM). Differential expression of the experimental groups was analyzed by a two‐tailed Mann–Whitney rank sum test with the Benjamini–Hochberg false discovery rate (FDR) multiple testing correction method. Differences in miRNA or gene expression were evaluated by the Student's *t*‐test. Differences with *p* < 0.05 were considered statistically significant. To evaluate the potential of the different miRNAs to serve as biomarker classifiers of AL amyloidosis and MM, receiver operating characteristic (ROC) analysis was used, as described in the Appendix S1. R command (cor.test) was used to calculate the Spearman rank correlation coefficients between miR‐9‐5p, miR‐181‐5p, and BCL2 gene expression.

## 3 RESULTS

Bone marrow samples were collected from 73 pretreated patients with AL amyloidosis (median age 67 years, range 42–85) and 80 pretreated MM patients (median age 66 years, range 42–90). Patient characteristics are described in Table S1.

### 3.1 Comparison of miRNA expression patterns in AL amyloidosis and MM

**FIGURE 1:**
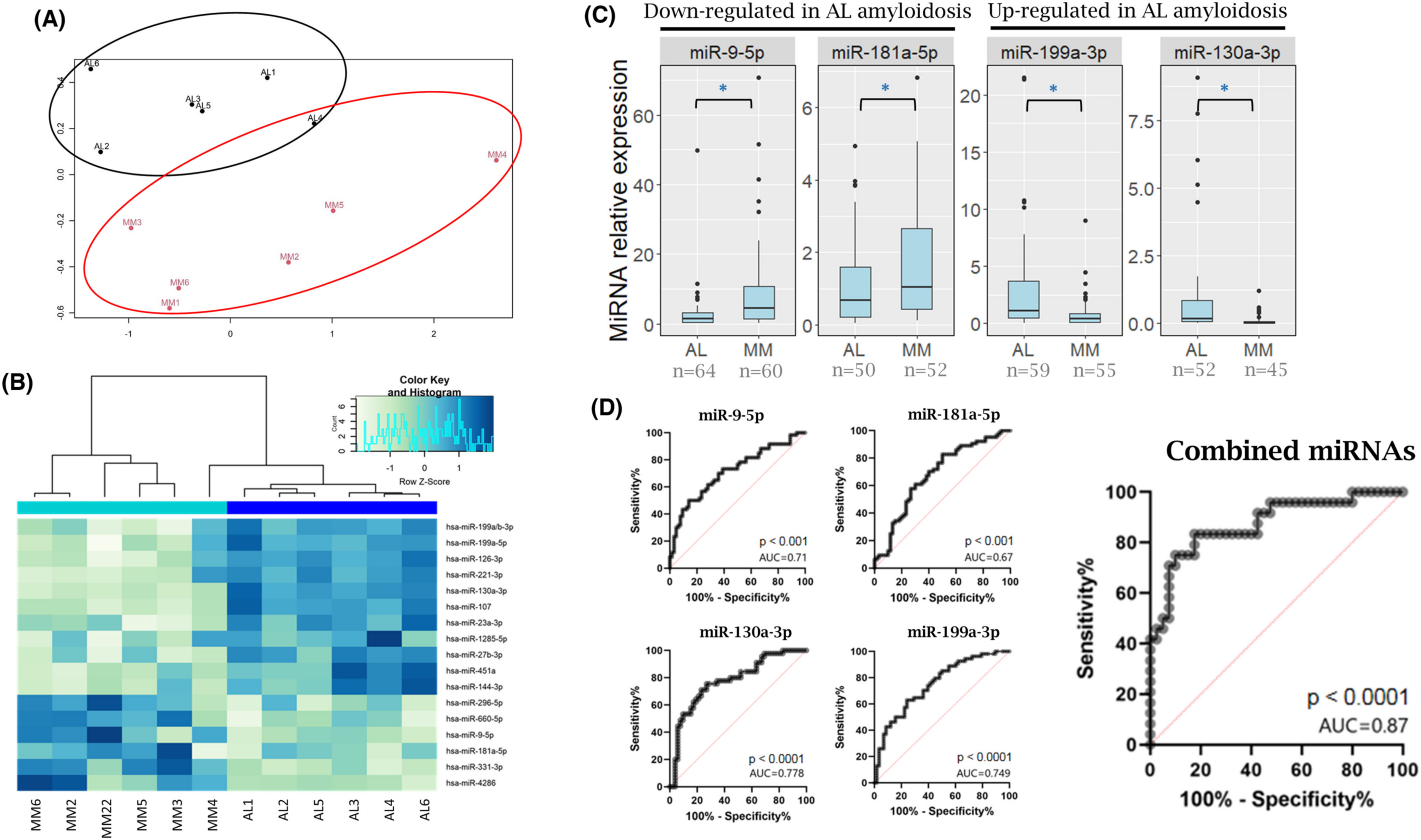
miRNA expression pattern in AL amyloidosis compared with MM. (A) A multidimensional scaling plot showing the differential expression of miRNAs from BM‐derived CD138+ cells of individuals with AL amyloidosis (black, *n* = 6) and MM (red, *n* = 6). (B) A heatmap of the normalized read counts of differentially expressed miRNAs in BM samples of individuals with MM and AL amyloidosis. Each row denotes a different miRNA, and each column denotes an individual patient sample. Green and blue and green represent the relatively low and high expression of miRNAs, respectively. (C) Validation of miRNA expression in an extended cohort of BM samples attained from individuals with AL amyloidosis (*n* ≥ 50) and MM (*n* ≥ 45) by qRT‐PCR normalized to spike‐in control cel‐miR‐39. (D) ROC curve showing the difference between AL amyloidosis and MM in the expression of miR‐9‐5p, miR‐130a‐5p, miR‐181a‐5p, miR‐199a‐3p, or a combined panel of the four miRNAs. AUC, area under the curve; BM, bone marrow; MM, multiple myeloma; qRT‐PCR, quantitative real‐time polymerase chain reaction; ROC, receiver‐operating characteristic. *p < 0.05

The expression of 800 known mature human miRNAs was initially analyzed in BM‐CD138+ cells obtained from six AL amyloidosis patients (AL1‐AL6) and six MM patients (MM2‐MM6 and M22). Principal component analysis (PCA) conducted for dimensionality reduction showed that AL amyloidosis patients' miRNAs were clustered separately from those of MM patients demonstrating a distinct miRNA expression pattern (Figure 1A). Seventeen miRNAs showed significant differential expression in AL CD138+ cells compared with MM CD138+ cells (six were downregulated and 11 were upregulated, FDR < 0.1; Figure 1B, Table S2).

To validate the miRNA NanoString data and to confirm that the observed variability was not technical, four BM miRNAs (miRs‐ 130a‐3p, 199a‐5p, 9‐5p, and 181a‐5p) were analyzed by TaqMan miRNA qRT‐PCR on at least 45 patient samples from each group (number of samples varied according to the availability of biological material). These validation of four miRNAs were chosen since they were among the miRNAs with the largest fold change between AL amyloidosis and MM and were previously shown to have a regulatory role in the pathogenesis of hematological malignancies^24^, ^25^ (Table S2). The qRT‐PCR validation showed similar results to those observed by the NanoString analysis (Figure 1C). There was no significant correlation between miRNA expression and each group's age (*p* > 0.05).

Receiver operating characteristic analysis was performed to assess the classification performance of these miRNA in distinguishing AL amyloidosis and MM. miR‐9‐5p had a 73.3% sensitivity and 60.9% specificity in discriminating between AL amyloidosis and MM, with an area under the curve (AUC) of 0.71 (*p* < 0.001, 95% CI: 0.62–0.8). The miR‐181a‐5p had an AUC of 0.67 (*p* < 0.001; sensitivity = 70.3%; specificity = 60%, 95% CI: 0.58–0.773) and miR‐199a‐3p had an AUC of 0.749 (*p* < 0.0001; sensitivity = 70.4%; specificity = 63.8; 95% CI: 0.659–0.838). The analysis of the individual miRNAs showed that miR‐130a‐3p expression had the highest AUC value (0.778, *p* < 0.0001; sensitivity = 75.5%; specificity = 73.1%; CI: 0.68–0.87). Combining these four miRNAs improved classification accuracy, increasing the value of the AUC to 0.87 (*p* < 0.0001, sensitivity = 83.3%; specificity = 82.5%, 95% CI: 0.78–0.966) (Figure 1D).

### 3.2 Comparison of mRNA expression patterns in AL amyloidosis and MM

To identify the coding gene expression signature, the RNA of BM‐derived CD138+ cells from four AL amyloidosis patients and four MM patients was sequenced.

**FIGURE 2:**
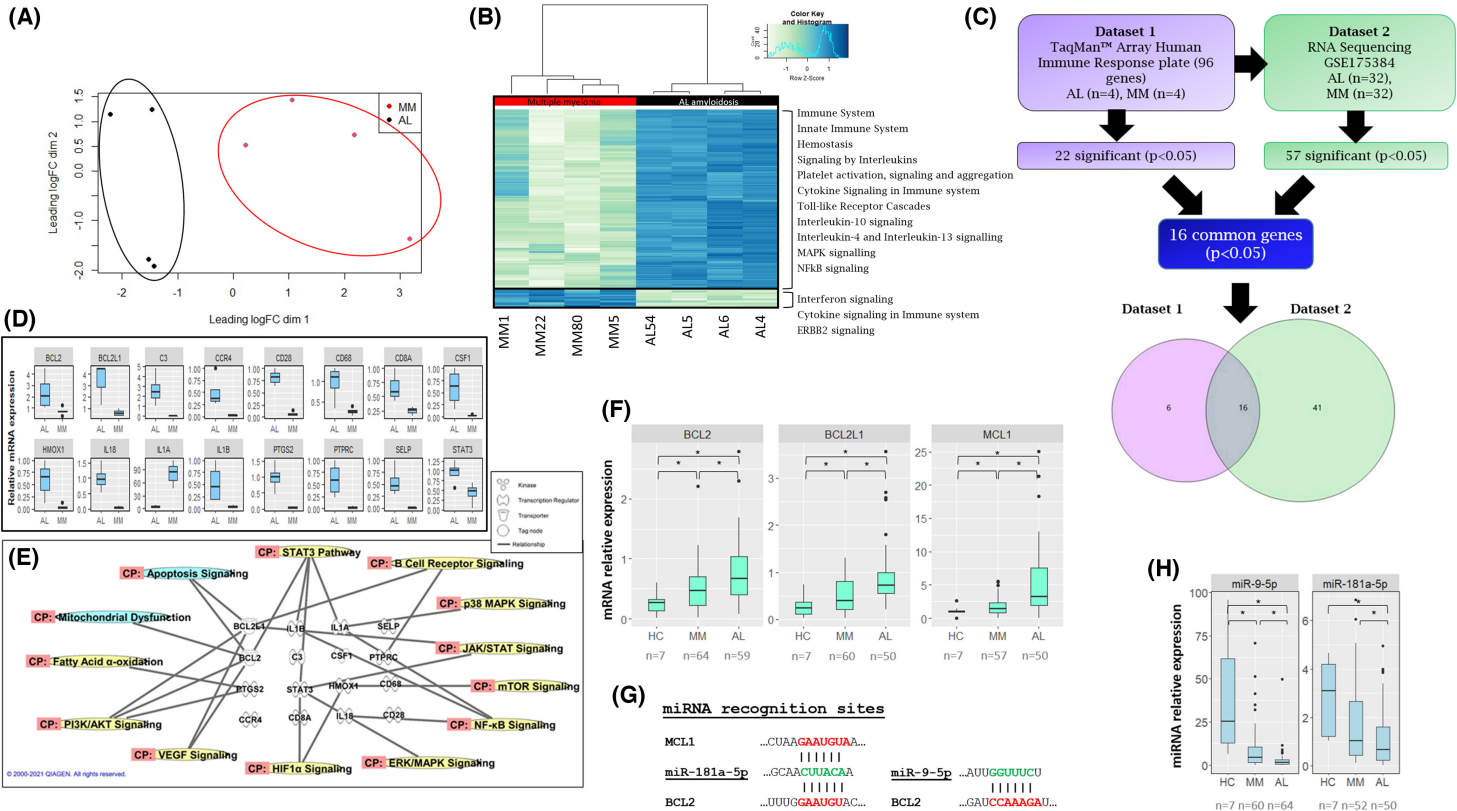
mRNA expression patterns in patients with AL amyloidosis compared with MM and gene expression pattern related to the human immune system. (A) A multidimensional scaling plot of the RNA sequencing data representing the distances corresponding to the differences in the biological coefficient of variation between BM samples of patients with MM (red) and AL amyloidosis (black). (B) A heat map illustrating unsupervised clustering of RNA expression in patients with MM (red) and AL amyloidosis (black). Blue and green indicate high and low expression, respectively. Gene ontology analysis of the genes that were differentially expressed demonstrated a potential involvement of these genes in regulating key signaling pathways as indicated on the right side of the heatmap. (C) Analysis of 96 genes by TaqMan™ Array Human Immune System plate revealed 22 significant genes (*p* < 0.05) differentiating patients with AL amyloidosis from patients with MM (Section 3.2.1). Comparison of these 96 genes to RNA sequencing data from the GEO database (GSE175384) showed that the expression of 57 of them was statistically significant (*p* < 0.05, Section 3.2.2). Sixteen genes were common to both analyses. (D) Graphs representing the 16 common BM genes that were statistically significantly expressed by qRT‐PCR between AL amyloidosis samples (*n* = 4) and MM samples (*n* = 4), (*p* < 0.05). GUSB and HPRT1 were used as endogenous controls for normalization. (E) Ingenuity pathway analysis showing the involvement of the 16 genes in regulating key biological pathways. (F) BCL2, MCL1, and BCL2L1 expression levels measured by qRT‐PCR (normalized to HPRT1 and GUSB endogenous controls) in BM‐derived CD138+ cell samples of patients with AL amyloidosis, MM, and HC. (G) Bioinformatics analysis, using TargetScan, of the predicted miR‐9‐5p and miR‐181a‐5p binding sites in the 3′UTR of the BCL2 and MCL1 genes. (H) miRs‐9‐5p and 181a‐5p expression levels measured by qRT‐PCR in BM‐derived CD138+ cell samples of patients with AL amyloidosis, MM, and HC. *p < 0.05. AL, AL amyloidosis; BM, bone marrow; MM, multiple myeloma; HC, healthy control; CP, canonical pathway; GUSB, glucuronidase beta; HPRT1, hypoxanthine phosphoribosyltransferase 1; qRT‐PCR, quantitative real‐time polymerase chain reaction

PCA showed that mRNAs of AL amyloidosis patients' were separately clustered from mRNAs of MM patients, demonstrating a distinct mRNA expression pattern (Figure 2A). One hundred sixty‐seven genes involved in various biological pathways were differentially expressed, specifically pathways related to the immune system, cell cycle regulation, mitogen‐activated protein kinase (MAPK), and nuclear factor kappa‐light‐chain enhancer of activated B cells (NFkB) (Figure 2B).

#### 3.2.1 Dataset 1 analysis

To validate the RNA‐seq results, we focused on one of the prominently enriched pathways observed in the RNA‐seq analysis: a subset of genes belonging to the immune system (Figure 2B). First, TaqMan™ Array Human Immune Response (Figure S1) was used to validate the findings in BM‐derived CD138+ cells from four AL amyloidosis patients and four MM patients. Among the 96 probes included in the qRT‐PCR plate, 67 genes were expressed in AL or MM samples (Figure S2). Among them, 22 genes were significantly differentially expressed (*p* < 0.05) between AL amyloidosis and MM patients (Figure S2, Figure 2C). Twenty‐six genes were not detected by qRT‐PCR in those samples (data not shown).

#### 3.2.2 Dataset II analysis

Dataset II was derived from a recent publication that examined the gene expression profile of AL amyloidosis and MM patients.^26^ Re‐analysis of the gene expression pattern from GSE175384 showed that the aberrantly expressed genes in AL amyloidosis and MM are strongly related to mitochondrial signaling pathways, specifically oxidative phosphorylation, sirtuin signaling pathway and mitochondrial dysfunction (Figure S3).

#### 3.2.3 Combined analysis datasets I + II

Next, we compared the available published data (GSE175384; e.g., Section 3.2.2) with the 96 gene signature we examined by qRT‐PCR (e.g., Section 3.2.1). Among the 96 genes, we found 16 common genes in both datasets (BCL2, BCL2L1, C3, CCR4, CD28, CD68, CD8A, CSF1, HMOX1, IL18, IL1A, IL1B, PTGS2, PTPRC, SELP, STAT3) that were significantly altered between AL amyloidosis and MM patients (*p* < 0.05; Figure 2C,D). Using IPA we found a possible involvement of these 16 genes in cell survival (e.g., JAK/STAT, mTOR, ERK/MAPK, and PI3K signaling pathways), mitochondrial dysfunction, and anti‐apoptotic mechanisms (Figure 2E). Since apoptosis signaling is mediated through the mitochondria, and since mitochondrial signaling pathways were prominent in dataset II (Figure S3), we further focused the analysis on these pathways.

### 3.3 BCL2 anti‐apoptotic family members are upregulated in AL amyloidosis

Among the above 16 overlapping genes that were significantly altered between CD138+ cells from AL amyloidosis and MM patients, BCL2 and BCL2L1 were highly expressed in AL amyloidosis compared with MM (Figure 2D). Therefore, we further analyzed the expression of BCL2, BCL2L1, and MCL1 (i.e., BCL2 family members) in an extended cohort of samples derived from BM of AL amyloidosis patients (range 57–64 samples), and MM patients (range 50–59) (depending on the availability of biological material) and compared them to the expression of these genes in BM samples attained from seven HCs who had hip replacement. The analysis confirmed that these anti‐apoptotic genes were upregulated in AL amyloidosis patients compared with MM patients and HCs (Figure 2F).

### 3.4 Regulation of the BCL2 family members by microRNAs

Since miRNAs regulate gene expression we used the bioinformatics tools IPA, miRTarBase, and TargetsScan, to explore a connection between the downregulated miRNAs in AL amyloidosis and the BCL2, BCL2L1, and MCL1 genes (Figure 2G). MiR‐181a‐5p was previously found, by luciferase assay, to directly affect the BCL2 and MCL1 genes,^27^ and miR‐9‐5p was shown to affect the expression of BCL2.^28^ These miRNAs were downregulated (Figure 2H), and BCL2, BCL2L1, and MCL1 genes were upregulated (Figure 2F) in samples from AL amyloidosis patients compared with MM and HC samples. BCL2 mRNA expression was found to be negatively correlated with miR‐9‐5p and miR‐181a‐5p (Spearman's correlation of −0.26 and −0.27, respectively, *p* < 0.05).

### 3.5 Upregulation of miR‐9 and miR‐181a‐5p in ALMC‐1 cells results in downregulation of BCL2, MCL1, and BCL2L1

**FIGURE 3:**
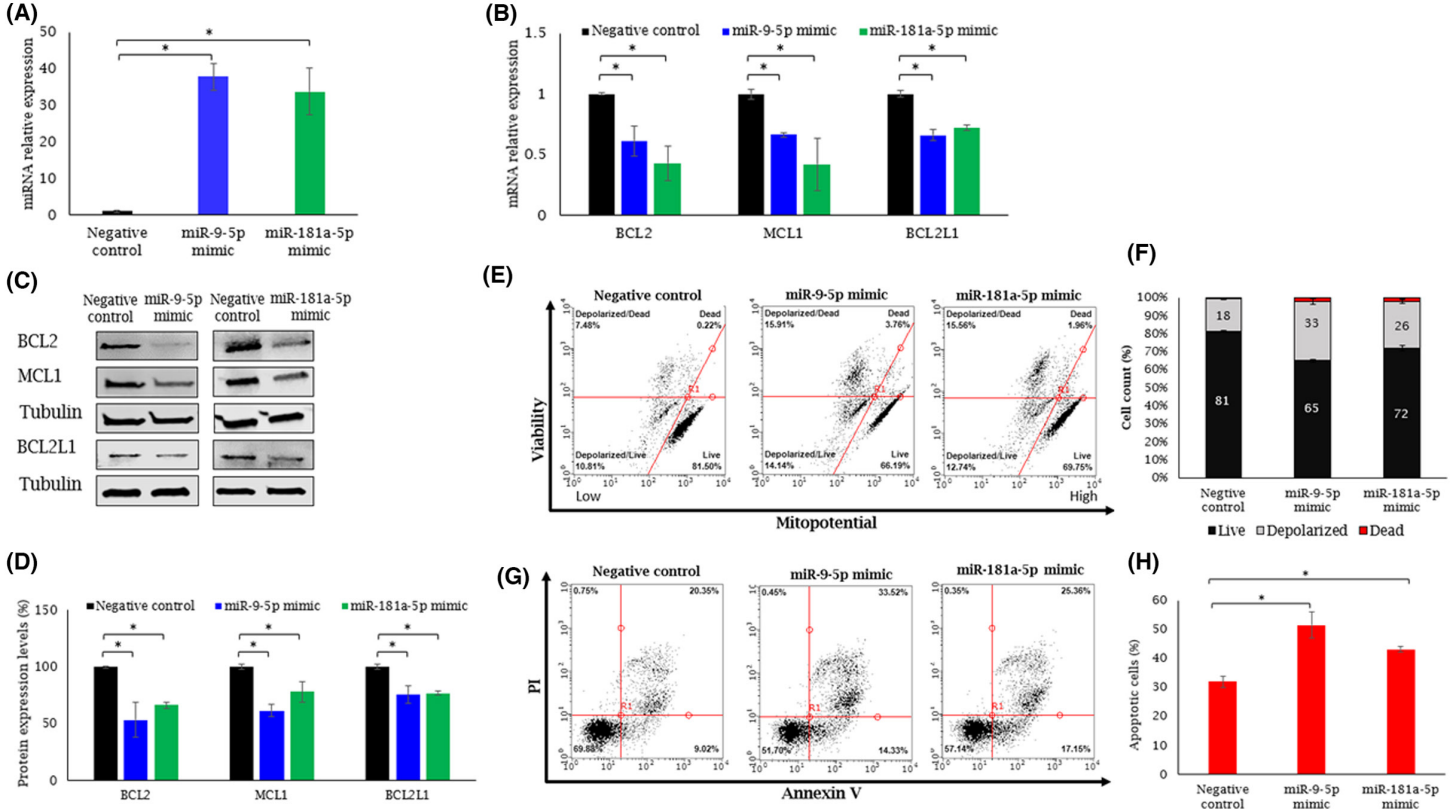
Upregulation of miR‐9 and miR‐181a‐5p in ALMC‐1 cells downregulated the BCL2, MCL1, and BCL2L1 genes and protein expression, and induces mitopotential activity and apoptosis in vitro. (A) ALMC‐1 cells were transfected with miR‐9‐5p and miR‐181a‐5p mimics (50 nM) or with a negative control for 48 h. The bars denote the mean expression of miR‐9‐5p and miR‐181a‐5p post‐transfection as measured by qRT‐PCR (normalized to snRNA U6) ± SEM. (B) Mean mRNA expression ± SEM from at least three experiments and (C) Western analysis showing BCL2, MCL1, and BCL2L1 protein levels after transfection with miR‐9‐5p or miR‐181a‐5p mimics. Tubulin was used as a loading control. (D) Mean protein levels ± SEM from at least three experiments of BCL2, MCL1, and BCL2L1 after transfection with miR‐9‐5p or miR‐181a‐5p mimics. (E) The percentages of 4 cell populations: live (mitopotential+/7‐AAD−), depolarized live (mitopotential−/7‐AAD−), dead (mitopotential+/7‐AAD+), and depolarized dead (mitopotential−/7‐AAD+) were measured using the Muse® Cell Analyzer. (F) The histogram values denote the mean of at least three experiments. Black, gray, and red denote the percentage of live cells, depolarized (live and dead) cells, and dead cells, respectively. (G) ALMC‐1 cells were stained with Annexin V and propidium iodide (PI) and analyzed using the Muse cell analyzer. (H) The histograms on the right denote Annexin V‐positive cells transfected with miR‐9‐5p or miR‐181a‐5p mimics or a negative control. The values are the mean of at least three experiments. *p < 0.05. qRT‐PCR, quantitative real‐time polymerase chain reaction; SEM, standard error of the mean

Next, we investigated whether miR‐9‐5p and miR‐181a‐5p upregulation affects the BCL2 anti‐apoptotic family members' expression. To that end, we transfected ALMC‐1 cells with miR‐9‐5p or miR‐181a‐5p mimics, which caused 30–40‐fold upregulation of these miRNAs (Figure 3A) followed by the downregulation of BCL2, MCL1, and BCL2L1 mRNAs (Figure 3B) and proteins (Figure 3C,D).

### 3.6 Upregulation of miR‐9 and miR‐181a‐5p in ALMC‐1 cells induced mitochondrial membrane potential depolarization and cell apoptosis

Mitochondrial membrane potential depolarization prevents the entry of calcium into the mitochondria and is a crucial step in the progression to cell death.^29^

To test whether miR‐9‐5p and miR‐181a‐5p have a role in this process, we examined the effect of overexpression of these miRNAs on mitochondrial potential and permeabilization of cellular plasma membrane.^29^ To that end, we transfected ALMC‐1 cells with miR‐9‐5p and miR‐181a‐5p mimics for 48 hours. Then, we determined the percentage of live cells with intact mitochondria, the percentage of depolarized live and dead cells, and the percentage of dead cells with intact mitochondria. Figure 3 panels E,F show that compared with the percentage of depolarized cells transfected with a scrambled miRNA control (18%), miR‐9‐5p and miR‐181a‐5p overexpression significantly increased the percentage of dead and live depolarized cells to 33% and 26%, respectively. Moreover, the overexpression of miRs‐9‐5p and 181a‐5p demonstrated a substantial increase in the rate of apoptotic ALMC‐1 cells measured by flow cytometry after staining with Annexin V and propium iodide (PI) (Figure 3G,H), suggesting a biological effect of miR‐9‐5p and miR‐181a‐5p in regulating BCL2 family members in AL amyloidosis. Interestingly, when we reanalyzed the gene expression pattern from GSE175384 dataset, we found significant expression of genes involved in mitochondrial dysfunction pathways (*p* < 0.002; Figure S3) with possible interactions between miR‐9‐5p, miR‐181a‐5p and anti‐apoptotic BCL2 family members (Figure S4)*,* as detected by IPA analysis.

### 3.7 Analysis of miR‐9‐5p, miR‐181a‐5p, and BCL2 family members' expression in AL amyloidosis and MM patients stratified by translocation 11:14 detected by FISH

Cumulative evidence shows promising results in treating MM patients with the BCL2 inhibitor venetoclax, particularly in those whose clonal plasma cells overexpress BCL2 and/or have translocation t(11;14).^30^, ^31^ In addition, data from AL amyloidosis are even more promising with the majority of relapsed/refractory AL patients with t(11;14) and show deep responses to venetoclax, with responses seen also among non‐t(11;14), albeit at lower proportion.^32^, ^33^ The expression of miR‐9‐5p, miR‐181a‐5p, BCL2, MCL1, and BCL2L1 in AL amyloidosis and MM patients was analyzed by t(11:14) status (which was determined by fluorescence in situ hybridization [FISH]). Although clinical data availability limitations resulted in a smaller sample size, we found no significant difference between the miRNAs (Figure S5A) or BCL2 (Figure S5B, left graph) expression levels and the presence of t(11:14), albeit a trend for lower miRNA expression was noted among AL amyloidosis patients in comparison with MM patients. Irrespective of t(11;14) status, BCL2 expression levels were not significantly different between AL amyloidosis and MM and by t(11;14) status (Figure S5B, left panel), while MCL1 expression was significantly higher in AL amyloidosis patients compared with MM patients when stratified by t(11;14) (Figure S5B, middle panel). By contrast, BCL2L1 expression was lower in AL amyloidosis and MM patients harboring t(11:14) compared with patients without this translocation (Figure S5B, right panel). Next, we calculated the BCL2/BCL2L1 ratio and the BCL2/MCL1 ratio in patients with and without t(11:14). As previously reported,^34^ the ratio between BCL2/BCL2L1 was significantly higher in MM patients with t(11:14) compared with MM patients without t(11:14) (*p* < 0.05), but this observation was not found in AL (Figure S5C). The BCL2/MCL1 ratio was not affected by t(11:14) in either AL amyloidosis or MM (Figure S5C).

### 3.8 Treatment with venetoclax affects miRNAs 9‐5p and 181a‐5p and BCL2 family members expression levels and induce apoptosis in ALMC‐1 cells

**FIGURE 4:**
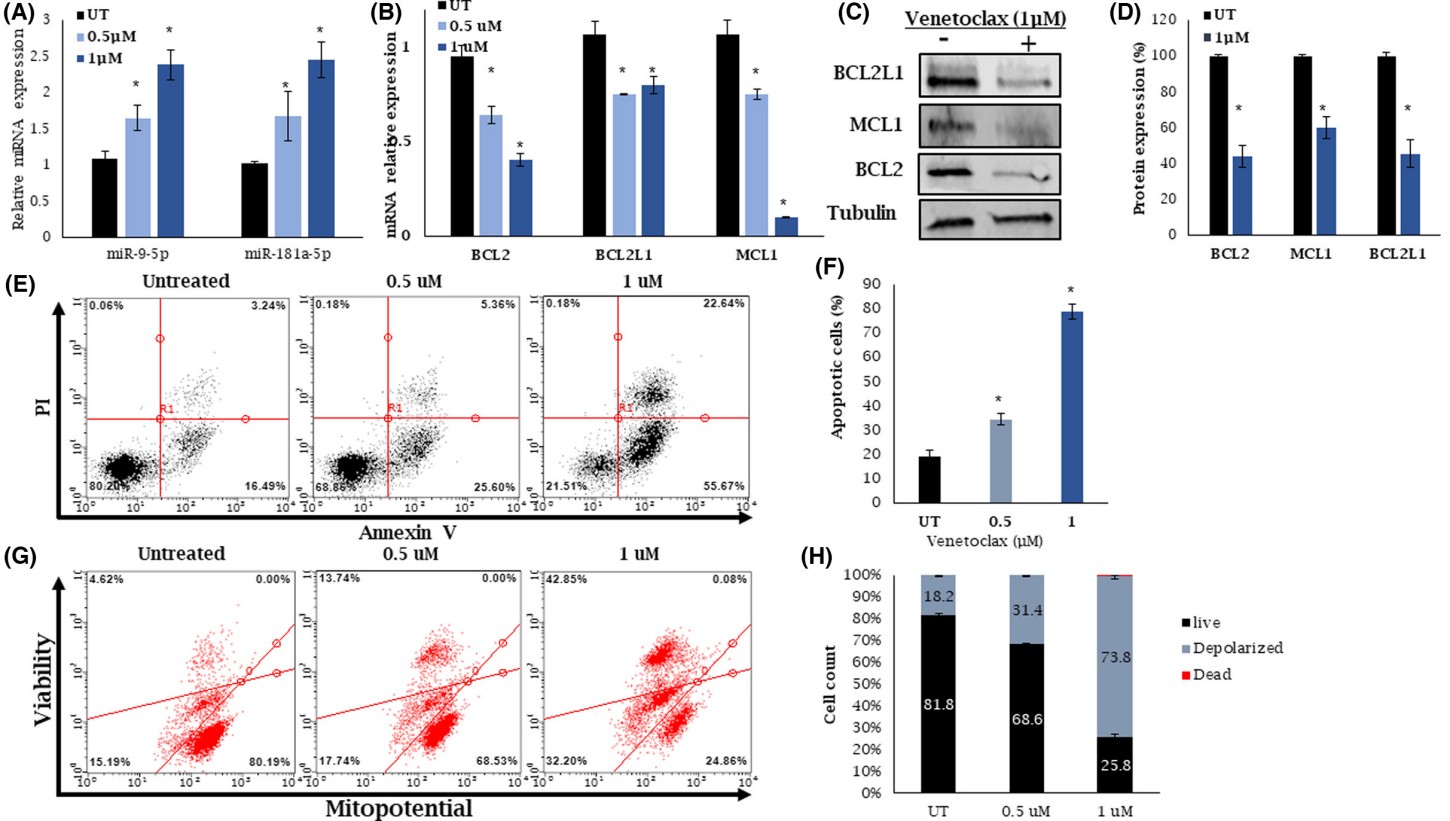
Treatment with venetoclax effect on miRNA, mRNA, protein levels and induced apoptosis and mitochondrial depolarization in ALMC‐1 cells. (A) Expression analysis of miR‐9‐5p and miR‐181a‐5p and (B) BCL2, BCL2L1, and MCL1 mRNA levels measured by qRT‐PCR before and following treatment with 0.5 and 1 μM venetoclax ± SEM from at least three experiments. (C) Western blot analysis showing BCL2, MCL1, and BCL2L1 protein levels after treatment with 1 μM venetoclax. Tubulin was used as a loading control. (D) Protein expression values are the mean of at least three experiments ± SEM. (E) ALMC‐1 cells were treated with 0.5 and 1 μM venetoclax, stained with Annexin V and propidium iodide (PI), and then analyzed by Muse® Cell Analyzer. (F) The histograms represent the mean of apoptotic cells + SEM from at least three experiments. (G) The percentages of 4 cell populations: live (mitopotential+/7‐AAD−), depolarized live (mitopotential−/7‐AAD−), dead (mitopotential+/7‐AAD+), and depolarized dead (mitopotential−/7‐AAD+) were measured using the Muse® Cell Analyzer following treatment with 0.5 and 1 μM venetoclax. (H) The histogram values are the mean mitochondrial potential of at least three experiments. Black, gray, and red represent the percentage of live cells, depolarized (live and dead) cells, and dead cells, respectively. *p < 0.05. SEM, standard error of the mean; UN, untreated

Since BCL2 expression was higher in AL patients' samples compared with MM and HC, we tested the sensitivity of ALMC‐1 cells to the BCL2 inhibitor venetoclax. The cells were subjected to increasing doses of venetoclax (0–10 μM) for 48 h (Figure S6). Concentrations of 0.5 and 1 μM were used for all subsequent experiments. Treatment of ALMC‐1 cells with venetoclax, showed the upregulation of miRNA‐9‐5p and miRNA‐181a‐5p expression (Figure 4A) followed by the downregulation of BCL2, MCL1, and BCL2L1 mRNA (Figure 4B) and protein levels (Figure 4C,D). Finally, treatment with venetoclax induced apoptosis and increased mitochondrial depolarization (Figure 4E–H) of ALMC‐1 cells.

## 4 DISCUSSION

This study is one of few studies that examined the differences in the gene expression profile between AL and MM. Evaluation of BM‐derived plasma cells from individuals with AL amyloidosis or MM and from HC, showed that AL cells strongly depend on the anti‐apoptotic BCL‐2 family proteins (BCL2, BCL2L1, and MCL1), regardless of t(11;14) status. These genes have multiple roles, including immune response modulation, cell growth, and apoptosis regulation.^35^, ^36^ Overexpression of anti‐apoptotic BCL2 family members contributes to the development of hematological malignancies including MM,^37^, ^38^ CLL,^39^ acute myeloid leukemia,^40^ and non‐Hodgkin's lymphoma.^41^ However, to the best of our knowledge, their expression in a large cohort of individuals with AL amyloidosis has not been determined to date. The development of small‐molecule inhibitors to the main anti‐apoptotic proteins of the BCL‐2 family has led to target abnormal cell populations as a therapeutic opportunity.

We have demonstrated that miR‐9‐5p and miR‐181a‐5p were downregulated in BM‐derived CD138+ cells from individuals with AL amyloidosis compared to those with MM and HCs. This downregulation is likely a key factor in the upregulation of the BCL2 gene members. Overexpression of these miRNAs in ALMC‐1 cells resulted in BCL2, MCL1, and BCL2L1 downregulation and increased apoptosis. These findings imply that miR‐9‐5p and miR‐181a‐5p may be tumor suppressors in AL amyloidosis by regulating oncogenic BCL2 family members, demonstrating yet another level of post‐transcriptional regulation in this disease. Moreover, we observed the in vitro upregulation of these miRNAs followed by the downregulation of the BCL2 family members after treatment with venetoclax, suggesting that venetoclax has a dual effect on BCL2 expression (1) it directly inhibits its expression and (2) resulting in the upregulation of miRNAs that regulate the anti‐apoptotic proteins, which supports the use of BCL2 inhibitors in this disease.

Low miR‐9‐5p expression in acute lymphoblastic leukemia (ALL) was related to hypermethylation of the MIR9 gene family. This epigenetic downregulation results in the upregulation of fibroblast growth factor receptor 1 (FGFR1) and CDK6, which are involved in the proliferation of cells and their survival.^42^, ^43^ FGFR1 and CDK6 inhibitors suppress ALL cell proliferation.^44^ MIR9 genes are frequently methylated in chronic lymphocytic leukemia (CLL) as well. Overexpression of miR‐9‐5p decreased CLL cell proliferation.^45^ These suggest that hypermethylation might be involved in the downregulation of miR‐9‐5p in AL amyloidosis, which should be further explored. MiR‐181a‐5p negatively regulates the signaling of nuclear factor κ–light‐chain enhancer of activated B cells (NF‐κB) in diffuse large B‐cell lymphoma (DLBCL). Overexpression of miR‐181a‐5p significantly decreases key NF‐κB signaling components' expression and activity.^46^ As miR‐181a‐5p expression is downregulated in AL amyloidosis, it will be interesting to further explore whether NF‐κB pathway is involved in disease pathogenesis.

Although not all MM patients with t(11;14) show high BCL2 expression, it remains unclear why t(11;14) patients have BCL2 dependence. We found similar results to those reported by Cleynen et al.,^34^ Kumar et al.,^31^ and Kitadate et al.^47^ who demonstrated that MM patients with t(11:14) have a higher BCL2/BCL2L1 ratio. In our study, although AL amyloidosis patients with t(11:14) had a higher BCL2/BCL2L1 ratio compared with patients that did not harbor this translocation, this difference was not statistically significant, probably due to the small number of patients analyzed (*n* = 20 for patients with t(11:14) and *n* = 17 for those without the translocation). The above studies suggest that a high BCL2/BCL2L1 mRNA ratio correlates with response to venetoclax in MM patients, indicating the importance of examining not only BCL2 but rather the ratio between BCL2 and BCL2L1 in AL patients with t(11;14) in order to decide on treatment strategies.

Furthermore, in accordance with Cleynen et al.,^34^ no significant difference in the BCL2/MCL1 gene ratio was found between patients with AL amyloidosis harboring the t(11:14) and those that did not have this translocation.

AL‐derived plasma cells are more susceptible to cellular stress induced by the intrinsic toxicity of the amyloidogenic light chains.^48^ This includes defective autophagy and increases in the abundance of perinuclear mitochondria. Our data support the increase in mitochondria‐derived anti‐apoptosis pathways to overcome this intrinsic cellular stress. Although t(11;14) has been associated with an increase in BCL2 family member upregulation to support mitochondria function and cellular survival, our data may suggest that other mechanisms exist to upregulate mitochondria‐derived anti‐apoptosis pathways. Overall, these cellular events potentially explain the low clonal load with low proliferation that is typical for this disease,^3^ while maintaining anti‐apoptosis mechanisms to maintain hemostasis.

Finally, miRNAs may be biomarker candidates to aid in the diagnosis and classification of AL amyloidosis, MM, and potentially other plasma cell disorders. AL amyloidosis is closely related to MM; however, each has its own unique disease characteristics. We hypothesized that both diseases have a shared miRNA signature related to the aberrant plasma clone, in parallel to a unique miRNA signature related to the distinctive characteristics of each disease. The differential expression of miRs‐9a‐5p, 181a‐5p, 199a‐3p, and 130a‐3p between AL amyloidosis and MM, suggests their potential use as biomarkers for distinguishing between these conditions, particularly when combined together. This may help in therapeutic decisions if a distinction between AL and MM will be found to have therapeutic implications such as duration of therapy, preferred agents of choice, etc; however, this finding should be further validated in a larger multi‐center cohort of patients, including in other plasma cell disorders.

The study has several limitations. First, miRNA/gene expression was analyzed based on biological material availability, and therefore, the sample size is not equal among all experiments. Second, clinical data, specifically data regarding FISH abnormalities was unavailable for some patients, resulting in underpowered and unbalanced analysis. Moreover, although we did not find significant differential expression in miR‐9‐5p and miR‐181a‐5p when comparing t(11;14) status, other miRNAs might be influenced by the presence of this translocation, which should be explored in future studies. Third, as BM samples from HCs contains a very small amount of CD138+ cells, we could not sequence CD138+ cell population from HCs for miRNA or gene expression and only miR‐9‐5p, miR‐181a‐5p, BCL2, MCL1, and BCL2L1 were measured in those samples by qRT‐PCR, in which we could use a very small amount of RNA as input. Lastly, we lack comparative analysis to other plasma cell disorders, particularly MGUS and SMM.

In conclusion, miR‐9‐5p and miR‐181a‐5p act as tumor suppressors in AL amyloidosis. These miRNAs are downregulated, allowing the overexpression of BCL2 family members and the upregulation of anti‐apoptotic mechanisms. This study highlights the post‐transcriptional regulation in AL amyloidosis and proposes that specific miRNAs have therapeutic and diagnostic potential in the management of this disease.

## AUTHOR CONTRIBUTIONS

**Hila Fishov:** Conceptualization (equal); data curation (equal); formal analysis (equal); investigation (equal); writing – original draft (equal). **Eli Muchtar:** Conceptualization (equal); data curation (equal); formal analysis (equal); investigation (equal); methodology (equal); resources (equal); writing – original draft (equal). **Mali Salmon‐Divon:** Conceptualization (equal); data curation (equal); formal analysis (equal); methodology (equal); software (equal); visualization (equal); writing – original draft (equal). **Angela Dispenzieri:** Conceptualization (equal); methodology (equal); resources (equal); writing – original draft (equal). **Tal Zvida:** Investigation (equal); validation (equal); writing – review and editing (equal). **Claudio Schneider:** Resources (equal); writing – review and editing (equal). **Benjamin Bender:** Resources (equal); writing – review and editing (equal). **Adrian Duek:** Resources (equal); writing – review and editing (equal). **Merav Leiba:** Resources (equal); writing – review and editing (equal). **Ofer Shpilberg:** Conceptualization (equal); data curation (equal); investigation (equal); methodology (equal); resources (equal); writing – original draft (equal). **Oshrat Hershkovitz‐Rokah:** Conceptualization (equal); data curation (equal); formal analysis (equal); methodology (equal); writing – original draft (equal), Supervision; Funding acquisition.

## FUNDING INFORMATION

This work was supported by a research grant of the Israeli Society of Hematology and Blood Transfusion.

## CONFLICT OF INTEREST

Eli Muchtar: Protego (Consultancy, fee to institution), Janssen (Honararium). Angela Dispenzieri received research funding from Celgene, Millennium Pharmaceuticals, Pfizer, and Janssen and received a travel grant from Pfizer.

## ETHICS STATEMENT

The samples were collected at Mayo Clinic (Rochester, MN, USA) and Assuta Medical Centers (Tel‐Aviv and Ashdod, Israel) in accordance with the local Institutional Review Board protocols. All patients provided informed consent for their sample collection and medical chart review for research purposes.

## Supporting information


Appendix S1
Figure S1
Figure S2
Figure S3
Figure S4
Figure S5
Figure S6
Table S1
Table S2

## Data Availability

The RNA‐Seq raw reads were uploaded to NCBI (SRA accession no. PRJNA846139)
