# Supplementary material for: AL amyloidosis clonal plasma cells are regulated by microRNAs and dependent on anti‐apoptotic BCL2 family members
Source: Cancer Med. 2023 Jan 24;12(7):8199–210. doi: 10.1002/cam4.5621 (PMC10134277; doi:10.1002/cam4.5621)
Supplement: Supplementary file 1 — Appendix S1 Figure S1 Figure S2 Figure S3 Figure S4 Figure S5 Figure S6 Table S1 Table S2 [file CAM4-12-8199-s001.zip › CAM4_5621_Supplementary captions.docx]

**Supplementary Table 1. Patient's characteristics**

Abbreviations: C, White; B, Black; O, Other; P, Asian; U, Unk; A, American Indian; Organ involvement: H, Heart; K, kidney; L, Liver; N, Nerve; GI, Gastrointestinal; AB, Abnormal; N, Normal; NA, Not available.

**Supplementary Table 2. Up/Down regulated miRNAs in AL amyloidosis patients compare to multiple myeloma (NanoString analysis).**
