# Supplementary material for: AL amyloidosis clonal plasma cells are regulated by microRNAs and dependent on anti‐apoptotic BCL2 family members
Source: Cancer Med. 2023 Jan 24;12(7):8199–210. doi: 10.1002/cam4.5621 (PMC10134277; doi:10.1002/cam4.5621)
Supplement: Supplementary file 1 — Appendix S1 Figure S1 Figure S2 Figure S3 Figure S4 Figure S5 Figure S6 Table S1 Table S2 [file CAM4-12-8199-s001.zip › CAM4_5621_Supplementary files united .docx]

**Supplementary Material and Methods**

**Quantitative polymerase chain reaction (qRT-PCR)**

MiRNA was reverse-transcribed with miRNA-specific stem-looped RT primers (Life Technologies, Thermo Fisher Scientific Inc.) and incubated for 30 minutes at 16°C, 30 minutes at 42°C, and 5 minutes at 85°C. MiRNAs were detected using the TaqMan® Small-RNA primer and probe sets (Applied Biosystems, Thermo Fisher Scientific Inc.). Quantitative real-time PCR (qRT-PCR) was performed in duplicates using Step One Plus Real-Time PCR (Life Technologies, Thermo Fisher Scientific Inc.) under the following conditions: 95 °C for 20 seconds, followed by 40 cycles of 95 °C for 1 second, and 60 °C for 20 seconds. Each miRNA expression value was presented relative to the expression value of an external synthetic cel-miR-39 (Applied Biosystems, Thermo Fisher Scientific Inc.), which was used as an internal control. The fold change was calculated using the ΔΔCt method (Applied Biosystems™ Analysis Software, Relative Quantification Analysis Module, v4.1, Thermo Fisher Scientific Inc.).

For gene expression assays, RNA was reverse transcribed using the high-capacity cDNA RT kit (Life Technologies; Thermo Fisher Scientific Inc.). Complementary DNA was equally pre-amplified using the TaqMan® PreAmp Master Mix (Applied Biosystems, CA, USA) according to the manufacturer's protocol. Briefly, 12.5 μL cDNA were added to 50 μL PreAmp Master Mix together with 12.5 μL of the gene of interest and pre-amplified for 14 cycles. The samples were diluted 1:10 and TaqMan qRT-PCR was performed in duplicates using Step One Plus Real-Time PCR (Life Technologies; Thermo Fisher Scientific Inc.). Each reaction contained 30 ng DNA, 5 µl TaqMan master mix (Life Technologies, Thermo Fisher Scientific Inc.), and 1 µl fluorescein amidites (FAM)-labeled TaqMan probe of the target gene or glucuronidase beta (GUSB) and hypoxanthine phosphoribosyltransferase 1 (HPRT1) control primers (Life Technologies, Thermo Fisher Scientific Inc.). Reaction conditions for the gene expression assays were the same as for the miRNA qRT-PCR described above.

**Cell proliferation assay**

Cells (2*10^4/100µl) were treated with venetoclax. After 48h of incubation, 10 µl of WST-1 reagent (Roche, Basel, Switzerland) was added to the plate and incubated for 1h at 37°C, at which point the absorbance (450nm) was measured in an ELISA plate reader (Synergy HTX multi-mode reader, Winooski, Vermont, USA).

**Western analysis**

Proteins were separated by 10% sodium dodecyl sulphate–polyacrylamide gel electrophoresis (SDS-PAGE; Bio-Rad, USA) and blotted onto nitrocellulose membranes. The membranes were blocked and incubated with the following primary antibodies (all from Cell Signaling Technology, Danvers, MA USA): BCL2 (#4223), BCL2L1(#2764), MCL1(#94296) and tubulin (#3873). Fluorescent-labeled secondary antibodies were purchased from Li-Cor Biosciences (Lincoln, NE, USA). The membranes were scanned using LICOR FC Imaging System (Li-Cor Biosciences).

**Apoptosis**

The cells (2x10^5^; 65 µl) were stained with 65 µl Muse^®^ Annexin V and Dead Cell Reagent kit (Luminex, Austin, TX, USA) according to the manufacturer's instructions, incubated in the dark for 20 minutes at room temperature, and then analyzed using the Muse® Cell Analyzer system (Luminex, USA). The percentage of apoptotic cells was defined using the Guava Software version 3.3 (Luminex, USA).

**Receiver Operating Characteristic (ROC) analysis**
We performed ROC analysis using GraphPad Prism 9. To obtain the value of the combined miRNAs expression, we calculated a weighted average of the expression of the four miRNAs for each sample, in which the weights were equal to the Point-Biserial correlation of the expression of that miRNAs and the disease status across all samples. The p-value of the ROC curve tests the null hypothesis that the area under the curve equals 0.5. The cut-off points with the highest sensitivity and specificity were determined.

| **Supplementary Table 1.** | | | | | | | | | | | | | | | | | | | | |
| --- | --- | --- | --- | --- | --- | --- | --- | --- | --- | --- | --- | --- | --- | --- | --- | --- | --- | --- | --- | --- |
| **Sample ID** | **Age at sampling** | **Sex** | **Race** | **Organs involved** | **BM Plasma cells (%)** | **Serum Creatinine (mg/dL)** | **Serum Albumin (g/dL)** | **NT-proBNP (pg/mL)** | **TROPONIN T (ng/mL)** | **Light chain restriction** | **iFLC (mg/dL)** | **Chromose 13 abnormalities (deletion 13q, monosomy 13)** | **t(11:14)** | **del17p** | **t(4;14)** | **t(14;16)** | **t(14:20)** | **IgH translocation without identifibale partner** | **Del1p** | **Gain 1q** |
|  |  |  |  |  |  |  |  |  |  |  |  |  |  |  |  |  |  |  |  |  |
| AL1 | 55 | M | C | H | 20 | NA | 3.8 | NA | NA | LAMBDA | NA | NA | NA | NA | NA | NA | NA | NA | NA | NA |
| AL2 | 45 | F | C | H,K | 25 | 0.8 | 3.4 | 16451 | 0.03 | LAMBDA | 118 | AB | AB | N | N | N | N | AB | N | N |
| AL3 | 81 | M | C | H | 5 | 1.8 | 2.5 | 4584 | 0.02 | LAMBDA | 30.1 | NA | AB | NA | NA | NA | NA | NA | NA | NA |
| AL4 | 57 | M | C | H,N | 10 | 1.4 | 2.8 | 3321 | 0.01 | LAMBDA | 52.7 | N | AB | N | N | N | N | AB | NA | NA |
| AL5 | 70 | M | C | H,K | 15 | 1.6 | 3.5 | 7131 | 0.01 | LAMBDA | 46 | N | N | N | NA | NA | NA | N | N | N |
| AL6 | 70 | M | C | H,L,N | 20 | 1.5 | 2.5 | 1604 | NA | KAPPA | 154 | N | AB | N | NA | NA | NA | AB | N | N |
| AL7 | 79 | F | C | H,GI,N | 8 | 1.7 | 3 | 5334 | 0.03 | LAMBDA | 42.8 | N | N | AB | N | N | N | N | NA | NA |
| AL8 | 81 | F | C | H,L,GI | 70 | 0.9 | 3 | 769 | NA | LAMBDA | 31.9 | N | N | N | NA | NA | NA | N | N | AB |
| AL9 | 58 | M | C | H,K,L | 4.01 | 1.81 | 1.8 | 3587 | NA | LAMBDA | 20.3 | AB | AB | N | NA | NA | NA | AB | N | N |
| AL10 | 81 | M | C | None | 70 | 2.52 | 3.7 | NA | NA | KAPPA | 71.9 | AB | N | N | N | N | N | AB | NA | AB |
| AL11 | 64 | M | C | K,L,GI | 40 | 0.9 | 2.5 | 619 | 0.001 | KAPPA | 50.7 | N | N | N | N | N | N | AB | N | N |
| AL12 | 70 | M | C | H | 15 | 1.6 | 3.5 | 7131 | 0.01 | LAMBDA | 46 | N | N | N | NA | NA | NA | N | N | N |
| AL13 | 73 | M | C | H | 5 | 1.2 | 3.5 | 1153 | 0.02 | LAMBDA | 11.4 | N | AB | N | NA | NA | NA | AB | N | N |
| AL14 | 75 | M | C | H,K,N | 6 | 0.6 | 2.1 | 8576 | 0.02 | KAPPA | 61 | N | AB | N | N | N | N | N | NA | NA |
| AL15 | 73 | M | C | H,K,L | 22 | 1.3 | 3.1 | 12248 | 0.07 | LAMBDA | 8.03 | N | AB | N | N | N | NA | N | NA | NA |
| AL16 | 71 | F | C | H,K | 2 | 3 | 2.7 | 2195 | 0.01 | LAMBDA | 8.34 | N | N | N | N | N | NA | N | NA | NA |
| AL17 | 67 | F | C | H,GI | 8 | 1.3 | 3.2 | 19438 | 0.37 | KAPPA | 31.5 | AB | AB | N | N | N | NA | N | NA | NA |
| AL18 | 68 | M | C | H | 23 | 2 | 3.5 | 42800 | 0.31 | LAMBDA | 14.3 | AB | AB | N | N | N | NA | N | NA | NA |
| AL19 | 63 | M | O | H,K,N | 19 | 1 | 1.4 | 467 | 0.01 | LAMBDA | 49.4 | AB | N | N | N | N | NA | N | NA | NA |
| AL20 | 50 | M | C | H,K,L | 4 | 1.4 | 1 | 2899 | 0.08 | LAMBDA | 19 | N | AB | N | N | N | NA | N | NA | NA |
| AL21 | 70 | F | C | H,GI | 25 | 1.6 | 3.4 | 11462 | 0.17 | LAMBDA | 80.7 | AB | N | N | N | N | AB | AB | NA | NA |
| AL22 | 42 | F | C | H,L | 5 | 1.2 | 3.3 | 4927 | 0.1 | LAMBDA | 17 | N | N | N | N | AB | NA | N | NA | NA |
| AL23 | 75 | F | C | H,N | 20 | 0.92 | 3 | 6304 | NA | LAMBDA | 48.6 | N | AB | N | NA | NA | NA | NA | N | N |
| AL24 | 62 | F | C | H | 5 | 1.2 | 3.3 | 10287 | 0.31 | LAMBDA | 93.9 | AB | AB | N | N | N | N | AB | NA | NA |
| AL25 | 68 | M | P | H,K,L,N | 10 | 1.2 | 1.7 | 3509 | 0.02 | LAMBDA | 4.75 | N | AB | N | N | N | N | N | NA | NA |
| AL26 | 61 | M | C | H | 8 | 1.09 | 3.8 | 1032 | NA | LAMBDA | 57.2 | AB | N | N | NA | AB | NA | AB | N | N |
| AL27 | 67 | M | C | H | 10 | 1.3 | 3 | 38582 | 0.09 | LAMBDA | 48.8 | N | AB | N | NA | NA | NA | AB | N | N |
| AL28 | 59 | M | C | H,K,L,N | 4 | 0.7 | 1.6 | 2134 | 0.001 | LAMBDA | 31.3 | NA | N | NA | NA | NA | NA | NA | NA | NA |
| AL29 | 66 | M | C | H,K,L,GI | 12 | 0.9 | 1.4 | 462 | 0.04 | LAMBDA | 28.5 | AB | N | N | NA | NA | NA | AB | N | N |
| AL30 | 60 | M | C | H | 26 | 1.4 | 3.2 | 8459 | 0.24 | KAPPA | 244 | AB | N | NA | NA | NA | NA | N | N | N |
| AL31 | 53 | M | C | H,K,N | 4.01 | 1.2 | 2.7 | 6762 | 0.001 | LAMBDA | 41.8 | AB | AB | N | NA | NA | NA | AB | N | N |
| AL32 | 54 | M | C | H,N | 10.8 | 1.1 | 4 | NA | NA | LAMBDA | 6.79 | NA | NA | NA | NA | NA | NA | NA | NA | NA |
| AL33 | 65 | M | C | K | 9 | 1.15 | 1.6 | 406 | NA | LAMBDA | 10.1 | AB | AB | N | NA | NA | NA | AB | N | N |
| AL34 | 71 | M | C | H | 3 | 1.9 | 3.3 | 8282 | 0.1 | LAMBDA | 51.2 | NA | NA | NA | NA | NA | NA | NA | NA | NA |
| AL35 | 85 | M | C | H,N | 6 | 1.5 | 3.4 | 25977 | 0.07 | LAMBDA | 15.8 | NA | NA | NA | NA | NA | NA | NA | NA | NA |
| AL36 | 73 | M | B | H,K,N | 11 | 2.3 | 2.3 | NA | 0.18 | LAMBDA | 45.8 | NA | NA | NA | NA | NA | NA | NA | NA | NA |
| AL37 | 71 | M | C | None | 8.4 | 1.1 | 3.7 | 223 | 0.01 | KAPPA | 5.21 | NA | NA | NA | NA | NA | NA | NA | NA | NA |
| AL38 | 50 | M | C | H,K,GI | 13 | 1 | 1.3 | 523 | 0.08 | LAMBDA | 16.2 | NA | NA | NA | NA | NA | NA | NA | NA | NA |
| AL39 | 72 | M | C | H,GI,N | 19 | 1 | 3.3 | 3147 | 0.03 | LAMBDA | 6.73 | NA | NA | NA | NA | NA | NA | NA | NA | NA |
| AL40 | 46 | F | C | K | 9 | 1 | 1.8 | 374 | 0.01 | LAMBDA | 3.14 | NA | NA | NA | NA | NA | NA | NA | NA | NA |
| AL41 | 60 | M | U | None | 9 | 0.9 | 4.3 | 76.6 | 0.01 | KAPPA | 34.3 | NA | NA | NA | NA | NA | NA | NA | NA | NA |
| AL42 | 56 | F | C | H,N | 10 | 1.1 | 3.2 | 4038 | 0.1 | LAMBDA | 56.8 | NA | NA | NA | NA | NA | NA | NA | NA | NA |
| AL43 | 70 | M | C | H,K,L | 10 | 1.6 | 2.5 | 8859 | 0.06 | LAMBDA | 11.5 | NA | NA | NA | NA | NA | NA | NA | NA | NA |
| AL44 | 60 | F | C | H,K,L | 15 | 2.8 | 2.8 | 19921 | 0.49 | LAMBDA | 21 | NA | NA | NA | NA | NA | NA | NA | NA | NA |
| AL45 | 61 | M | C | K | 8 | 1 | 2.8 | 6.9 | 0.01 | LAMBDA | 20.8 | NA | NA | NA | NA | NA | NA | NA | NA | NA |
| AL46 | 54 | F | C | K | 3 | 0.5 | 2.6 | 94 | 0.001 | LAMBDA | 9.89 | NA | NA | NA | NA | NA | NA | NA | NA | NA |
| AL47 | 69 | M | O | H,K,N | 5 | 1.4 | 2.5 | 3171 | 0.001 | LAMBDA | 54.3 | NA | NA | NA | NA | NA | NA | NA | NA | NA |
| AL48 | 69 | M | C | H,K | 15 | 0.9 | 2.8 | 11842 | 0.08 | LAMBDA | 92.5 | AB | N | N | NA | NA | NA | N | N | AB |
| AL49 | 79 | M | C | None | 30 | 1.2 | 3.2 | 296 | 0.001 | KAPPA | 385 | AB | AB | N | NA | NA | NA | AB | N | AB |
| AL50 | 68 | M | C | H,K | 15 | 0.9 | 3 | 2416 | NA | LAMBDA | 7.68 | N | AB | N | NA | NA | NA | AB | N | N |
| AL51 | 65 | F | C | H,K,L,GI | 6 | 0.6 | 2.8 | 743 | NA | LAMBDA | 40.3 | AB | N | N | NA | NA | NA | AB | N | N |
| AL52 | 70 | M | C | H | 5 | 1.3 | 3.3 | 2180 | 0.03 | LAMBDA | 96.5 | N | AB | N | N | N | N | AB | NA | NA |
| AL53 | 78 | F | C | H,N | 15 | 0.8 | 3.1 | 4547 | 0.01 | LAMBDA | 38.3 | N | AB | N | N | N | NA | N | NA | NA |
| AL54 | 56 | F | C | None | 40 | 0.5 | 3.4 | 578 | 0.001 | KAPPA | 273 | AB | AB | N | NA | NA | NA | AB | N | AB |
| AL55 | 70 | M | C | H | 8 | 2.1 | 3.9 | NA | 0.2 | LAMBDA | 63.8 | NA | NA | NA | NA | NA | NA | NA | NA | NA |
| AL56 | 75 | F | C | H,K | 3 | 1.2 | 2.8 | 15730 | 0.05 | LAMBDA | 5.81 | NA | NA | NA | NA | NA | NA | NA | NA | NA |
| AL57 | 72 | M | C | H | 13 | 1 | 3.5 | 344 | 0.01 | KAPPA | 116 | NA | NA | NA | NA | NA | NA | NA | NA | NA |
| AL58 | 65 | F | C | H | 18 | 3.1 | 3 | 35000 | 0.45 | KAPPA | 271 | N | N | N | N | N | NA | N | NA | NA |
| AL59 | 66 | M | C | H | 3 | 2 | 3.3 | 1244 | 0.03 | KAPPA | 93.6 | NA | NA | NA | NA | NA | NA | NA | NA | NA |
| AL60 | 43 | F | C | H | 50 | 0.6 | 3.23 | 446 | 0.001 | KAPPA | 186 | AB | N | N | N | AB | N | AB | NA | AB |
| AL61 | 84 | M | C | H | 5 | 1.4 | 2.9 | 14258 | 0.22 | LAMBDA | 18.2 | N | AB | N | N | N | N | AB | NA | NA |
| AL62 | 66 | M | C | K,L | 24 | 1.3 | 2.4 | 273 | 0.001 | LAMBDA | 17.5 | AB | N | N | N | N | N | AB | NA | NA |
| AL63 | 79 | M | C | H | 43 | 1.3 | 3.4 | 9167 | 0.1 | LAMBDA | 25.8 | N | N | N | N | N | N | N | N | N |
| AL64 | 58 | M | C | K | 4.01 | 0.6 | 0.9 | 156 | 0.001 | LAMBDA | 25 | N | AB | N | N | N | N | AB | N | N |
| AL65 | 68 | F | C | H,N | 10 | 0.8 | 3.2 | 4006 | 0.001 | LAMBDA | 154 | N | AB | N | N | N | N | AB | N | AB |
| AL66 | 53 | F | C | H,N | 40 | 0.5 | 3.5 | 1992 | 0.03 | LAMBDA | 48 | AB | AB | N | N | N | N | AB | NA | NA |
| AL67 | 67 | F | C | H,L,GI | 7 | 1.2 | 2.9 | 7166 | 0.29 | KAPPA | 198 | AB | N | N | N | N | N | AB | N | N |
| AL68 | 72 | M | C | H | 20 | 0.9 | 2.6 | 20200 | 0.001 | KAPPA | 192 | AB | N | N | NA | NA | NA | AB | N | N |
| AL69 | 67 | M | C | H,K | 6 | 2.3 | 3.6 | 2556 | 0.12 | KAPPA | 79.3 | N | AB | N | NA | NA | NA | AB | N | N |
| AL70 | 66 | M | C | H,K | 50 | 0.9 | 3.7 | 10160 | 0.11 | LAMBDA | 316 | AB | AB | N | NA | NA | NA | AB | N | AB |
| AL71 | 70 | M | A | H,L | 10 | 1.5 | 2.2 | 23354 | NA | KAPPA | 63.9 | N | N | N | NA | NA | NA | N | N | N |
| AL72 | 68 | M | C | H | 20 | 1.6 | 3.8 | 11992 | 0.04 | KAPPA | 127 | N | AB | N | N | N | N | AB | N | N |
| AL73 | 49 | M | C | H,K | 5 | 0.8 | 1.7 | 2062 | 0.03 | LAMBDA | 21 | N | AB | N | NA | NA | NA | AB | N | N |
|  |  |  |  |  |  |  |  |  |  |  |  |  |  |  |  |  |  |  |  |  |
| MM1 | 54 | M | C |  | 9 | 0.9 | 3.9 | NA | NA | KAPPA |  | N | N | N | NA | NA | NA | N | N | N |
| MM2 | 81 | M | C |  | 60 | 1.7 | 3.2 | NA | NA | KAPPA |  | N | N | N | NA | NA | NA | N | N | N |
| MM3 | 66 | F | C |  | 70 | 0.9 | 3.9 | NA | NA | KAPPA |  | AB | N | N | NA | AB | NA | AB | N | AB |
| MM4 | 52 | M | C |  | 40 | 1 | 3.9 | NA | NA | KAPPA |  | N | N | N | NA | NA | NA | N | N | N |
| MM5 | 59 | M | C |  | 70 | 7.1 | 3.6 | NA | NA | KAPPA |  | NA | AB | AB | NA | NA | NA | AB | N | N |
| MM6 | 51 | M | C |  | 60 | 0.66 |  | NA | NA | KAPPA |  | NA | NA | NA | NA | NA | NA | NA | NA | NA |
| MM7 | 81 | M | C |  | 60 | 1 | 3.6 | 352 | 0.001 | KAPPA |  | N | AB | AB | NA | NA | NA | AB | N | AB |
| MM8 | 72 | M | C |  | 30 | 1.11 | 3.4 | 64 | NA | KAPPA |  | N | N | N | NA | NA | NA | AB | N | N |
| MM9 | 68 | F | C |  | 70 | 1.45 | 3.6 | 837 | NA | KAPPA |  | N | N | N | N | N | N | AB | N | N |
| MM10 | 77 | F | C |  | 60 | 1 | 3.4 | NA | NA | KAPPA |  | AB | N | N | NA | NA | NA | N | N | N |
| MM11 | 64 | F | C |  | 80 | 1.9 | 3.8 | NA | NA | KAPPA |  | N | AB | N | NA | NA | NA | AB | N | N |
| MM12 | 71 | F | C |  | 70 | 1.05 | 3.9 | NA | NA | KAPPA |  | N | AB | AB | NA | NA | NA | AB | NA | NA |
| MM13 | 75 | M | C |  | 33 | 1.5 | 3 | NA | 0.12 | KAPPA |  | N | N | N | N | N | NA | AB | NA | NA |
| MM14 | 60 | M | C |  | 80 | 1.8 | 3.2 | NA | NA | KAPPA |  | AB | N | N | NA | NA | NA | N | N | AB |
| MM15 | 70 | M | C |  | 71 | 1.24 | 3.3 | 170 | NA | LAMBDA |  | AB | N | N | NA | NA | NA | AB | N | AB |
| MM16 | 61 | F | C |  | 50 | 0.96 | 4 | NA | NA | KAPPA |  | N | AB | N | N | N | NA | AB | N | N |
| MM17 | 41 | M | C |  | 70 | 1.2 | 3.9 | 160 | NA | KAPPA |  | AB | AB | N | N | N | N | AB | NA | NA |
| MM18 | 45 | F | C |  | 93 | 0.7 | 3.9 | NA | NA | KAPPA |  | N | AB | N | N | N | NA | N | NA | NA |
| MM19 | 66 | M | C |  | 60 | 5.02 | 3.1 | NA | NA | LAMBDA |  | AB | N | N | NA | NA | NA | AB | AB | N |
| MM20 | 60 | M | C |  | 15 | 1.28 | 4 | NA | NA | KAPPA |  | AB | N | N | NA | NA | NA | N | N | AB |
| MM21 | 73 | M | C |  | 70 | 1.07 | 3.5 | 304 | NA | LAMBDA |  | N | N | N | NA | NA | NA | N | N | AB |
| MM22 | 67 | M | C |  | 86 | 1.4 | 3.56 | NA | NA | KAPPA |  | AB | N | N | N | N | NA | N | NA | NA |
| MM23 | 71 | F | C |  | 58 | 5.2 | 3.2 | NA | 0.11 | LAMBDA |  | N | N | N | N | N | NA | AB | NA | NA |
| MM24 | 73 | M | C |  | 50 | 1.3 | 3.8 | NA | NA | LAMBDA |  | AB | AB | N | N | N | NA | N | NA | NA |
| MM25 | 90 | F | C |  | 70 | 1.6 | 3.4 | NA | NA | LAMBDA |  | N | AB | AB | N | N | N | N | NA | NA |
| MM26 | 65 | M | C |  | 86 | 1 | 2.9 | NA | NA | KAPPA |  | AB | N | N | AB | N | NA | N | NA | NA |
| MM27 | 68 | F | C |  | 60 | 0.7 | 3.6 | NA | NA | LAMBDA |  | AB | N | N | N | N | NA | N | NA | NA |
| MM28 | 64 | F | P |  | 70 | 0.7 | 3.5 | 306 | NA | KAPPA |  | N | N | N | N | N | N | AB | N | N |
| MM29 | 50 | M | C |  | 60 | 1.08 | 4 | 592 | NA | KAPPA |  | AB | N | N | N | N | N | AB | N | N |
| MM30 | 55 | F | C |  | 10 | 2.5 | 3.1 | 648 | 0.001 | LAMBDA |  | N | AB | N | N | N | N | AB | NA | NA |
| MM31 | 59 | F | C |  | 30 | 0.6 | 3.9 | NA | NA | KAPPA |  | AB | N | N | N | N | N | N | N | AB |
| MM32 | 66 | M | C |  | 50 | NA | 3.9 | NA | NA | KAPPA |  | N | AB | N | NA | NA | NA | AB | N | AB |
| MM33 | 50 | M | C |  | 15 | 1 | 4.1 | 98 | 0.001 | KAPPA |  | AB | AB | N | NA | NA | NA | AB | N | N |
| MM34 | 56 | M | C |  | 30 | 4.4 | 3.7 | NA | NA | KAPPA |  | N | AB | N | NA | NA | NA | AB | N | N |
| MM35 | 71 | M | C |  | 30 | 0.9 | 3.6 | NA | NA | KAPPA |  | AB | N | AB | NA | NA | NA | N | N | N |
| MM36 | 62 | M | C |  | 75 | 3.2 | 4 | NA | NA | KAPPA |  | N | AB | N | NA | NA | NA | AB | N | N |
| MM37 | 80 | M | C |  | 20 | 0.95 | 3.6 | NA | NA | LAMBDA |  | N | N | N | NA | NA | NA | N | N | AB |
| MM38 | 76 | F | C |  | 80 | 1.35 | 3.6 | NA | NA | KAPPA |  | N | N | N | N | N | N | AB | N | N |
| MM39 | 68 | F | B |  | 80 | 0.84 | 2.9 | NA | NA | KAPPA |  | N | N | N | NA | NA | NA | N | N | N |
| MM40 | 55 | M | C |  | 3 | NA | 3.6 | NA | NA | NA |  | NA | NA | NA | NA | NA | NA | NA | NA | NA |
| MM41 | 67 | F | C |  | 70 | 0.81 | 3.8 | NA | NA | LAMBDA |  | AB | NA | AB | NA | AB | NA | NA | N | AB |
| MM42 | 71 | M | C |  | 80 | 1.4 | 3.3 | NA | NA | LAMBDA |  | NA | NA | NA | NA | NA | NA | NA | NA | NA |
| MM43 | 71 | M | C |  | 65.8 | 1.3 | 3.8 | NA | NA | KAPPA |  | NA | NA | NA | NA | NA | NA | NA | NA | NA |
| MM44 | 54 | M | C |  | 40 | 1.3 | 3.7 | NA | NA | LAMBDA |  | NA | NA | NA | NA | NA | NA | NA | NA | NA |
| MM45 | 49 | M | C |  | 80 | 3.2 | 3.6 | NA | NA | KAPPA |  | N | N | N | N | N | N | AB | AB | AB |
| MM46 | 59 | F | C |  | 20 | 0.8 | 3.8 | NA | NA | KAPPA |  | AB | N | N | N | N | N | AB | AB | AB |
| MM47 | 61 | M | C |  | 60 | 0.9 | 3.5 | NA | NA | KAPPA |  | AB | N | N | NA | NA | NA | AB | N | N |
| MM48 | 74 | F | C |  | 20 | 2.15 | 3.7 | 657 | NA | KAPPA |  | AB | AB | N | NA | NA | NA | AB | N | N |
| MM49 | 83 | F | C |  | 46 | 2.3 | 3.4 | NA | NA | KAPPA |  | NA | N | N | N | N | N | N | AB | NA |
| MM50 | 64 | M | C |  | 24 | 1.1 | 3.6 | 66 | 0.001 | KAPPA |  | NA | AB | N | N | N | N | N | N | NA |
| MM51 | 89 | M | C |  | 80 | 1.4 | 3.3 | 709 | 0.01 | LAMBDA |  | NA | AB | N | N | NA | NA | NA | AB | NA |
| MM52 | 63 | F | X |  | 10 | 0.9 | 3.7 | 36 | 0.001 | LAMBDA |  | AB | AB | N | NA | NA | NA | NA | N | N |
| MM53 | 76 | F | C |  | 25 | NA | 3.5 | NA | NA | LAMBDA |  | N | AB | N | NA | NA | NA | AB | N | N |
| MM54 | 85 | F | C |  | 10 | NA | 3.7 | NA | NA | KAPPA |  | N | N | N | NA | NA | NA | N | N | N |
| MM55 | 44 | M | C |  | 39 | NA | 3.8 | NA | NA | KAPPA |  | N | N | N | NA | NA | NA | N | N | N |
| MM56 | 81 | F | C |  | 70 | 0.9 | 3 | 769 | NA | LAMBDA |  | N | N | N | NA | NA | NA | N | N | AB |
| MM57 | 74 | M | C |  | 50 | 1.19 | 2.9 | NA | NA | LAMBDA |  | N | N | N | NA | NA | NA | N | N | N |
| MM58 | 47 | F | C |  | 43 | 0.9 | 4.2 | NA | NA | KAPPA |  | AB | AB | N | N | N | N | AB | NA | NA |
| MM59 | 75 | M | C |  | 97 | 1.3 | 3.3 | NA | NA | LAMBDA |  | AB | AB | AB | N | N | NA | N | NA | NA |
| MM60 | 47 | M | C |  | 12 | 1 | 3.9 | NA | NA | KAPPA |  | N | N | N | NA | NA | NA | N | N | N |
| MM61 | 70 | F | C |  | 60 | 0.7 | 4.2 | NA | NA | KAPPA |  | N | N | N | NA | NA | NA | N | N | N |
| MM62 | 51 | M | C |  | 60 | 1 | 3.6 | NA | NA | KAPPA |  | AB | N | N | AB | NA | NA | AB | N | AB |
| MM63 | 62 | F | C |  | 50 | 0.9 | 3.6 | 1885 | 0.001 | KAPPA |  | AB | N | N | NA | NA | AB | AB | N | AB |
| MM64 | 71 | M | O |  | 40 | NA | 3.9 | NA | NA | LAMBDA |  | N | AB | N | NA | NA | NA | AB | N | N |
| MM65 | 74 | M | C |  | 26 | 2.7 | 3.1 | NA | NA | LAMBDA |  | NA | NA | NA | NA | NA | NA | NA | NA | NA |
| MM66 | 62 | F | C |  | 30 | 0.8 | 3.6 | NA | NA | LAMBDA |  | N | AB | N | NA | NA | NA | AB | N | AB |
| MM67 | 71 | F | C |  | 41.8 | 1 | 3.4 | 1574 | 0.001 | KAPPA |  | AB | AB | N | N | N | N | AB | NA | NA |
| MM68 | 59 | M | C |  | 30 | 0.9 | 3.7 | NA | NA | KAPPA |  | AB | N | AB | N | N | N | AB | N | N |
| MM69 | 61 | M | C |  | 15 | NA | 3.4 | NA | NA | KAPPA |  | AB | N | NA | NA | NA | NA | N | N | N |
| MM70 | 60 | F | C |  | 60 | 0.4 | 3.9 | NA | NA | KAPPA |  | N | N | N | N | N | N | N | N | N |
| MM71 | 61 | M | P |  | 70 | 1.7 | 4.2 | NA | NA | LAMBDA |  | AB | N | N | AB | N | N | AB | NA | NA |
| MM72 | 63 | F | C |  | 50 | 0.9 | 3.9 | NA | 0.001 | KAPPA |  | N | N | N | N | N | N | N | NA | NA |
| MM73 | 71 | M | C |  | 20 | 1.1 | 3.8 | NA | NA | KAPPA |  | AB | N | N | N | N | N | N | NA | NA |
| MM74 | 67 | M | C |  | 40 | 0.8 | 3.6 | NA | NA | KAPPA |  | N | N | N | N | N | N | AB | N | AB |
| MM75 | 65 | M | C |  | 30 | NA | 3.9 | NA | NA | KAPPA |  | NA | N | N | NA | NA | NA | NA | NA | NA |
| MM76 | 55 | M | C |  | 50 | 1 | 3.9 | NA | NA | LAMBDA |  | N | N | N | NA | NA | NA | N | N | AB |
| MM77 | 77 | M | C |  | 30 | 1.3 | 3.9 | NA | NA | KAPPA |  | N | N | N | NA | NA | NA | N | NA | N |
| MM78 | 69 | F | O |  | 10 | NA | 2.6 | NA | NA | LAMBDA |  | NA | NA | NA | NA | NA | NA | NA | NA | NA |
| MM79 | 50 | M | C |  | 90 | 1.1 | 3.6 | NA | NA | LAMBDA |  | N | AB | N | N | AB | N | N | NA | NA |
| MM80 | 71 | M | C |  | 60 | 1.2 | 3.2 | NA | NA | LAMBDA |  | N | N | N | N | N | N | N | NA | NA |

**Supplementary Table 1. Patient's characteristics**

Abbreviations: C, White; B, Black; O, Other; P, Asian; U, Unk; A, American Indian; Organ involvement: H, Heart; K, kidney; L, Liver; N, Nerve; GI, Gastrointestinal; AB, Abnormal; N, Normal; NA, Not available.

**Supplementary Table 2.**

| **MiRNA ID** | **Log2 Fold Change** | **P value** | **P adjust** |
| --- | --- | --- | --- |
| hsa-miR-4286 | -2.56174 | 0.01077 | 0.066 |
| hsa-miR-9-5p | -2.497 | 0.008983 | 0.062 |
| hsa-miR-181a-5p | -1.78764 | 0.001041 | 0.014 |
| hsa-miR-660-5p | -1.66476 | 0.003054 | 0.026 |
| hsa-miR-296-5p | -1.19153 | 0.009665 | 0.063 |
| hsa-miR-331-3p | -1.16773 | 0.003119 | 0.026 |
| hsa-miR-23a-3p | 1.083563 | 0.017083 | 0.099 |
| hsa-miR-27b-3p | 1.154674 | 0.002928 | 0.026 |
| hsa-miR-1285-5p | 1.189711 | 0.000974 | 0.014 |
| hsa-miR-107 | 1.529153 | 0.000141 | 0.003 |
| hsa-miR-199a-5p | 2.024579 | 9.17E-05 | 0.003 |
| hsa-miR-221-3p | 2.035063 | 0.000103 | 0.003 |
| hsa-miR-144-3p | 2.208396 | 0.002699 | 0.026 |
| hsa-miR-199a-3p +  hsa-miR-199b-3p | 2.485758 | 0.000176 | 0.003 |
| hsa-miR-451a | 2.645121 | 0.002859 | 0.026 |
| hsa-miR-126-3p | 3.032426 | 0.000178 | 0.003 |
| hsa-miR-130a-3p | 3.251533 | 1.94E-07 | 2.15E-05 |


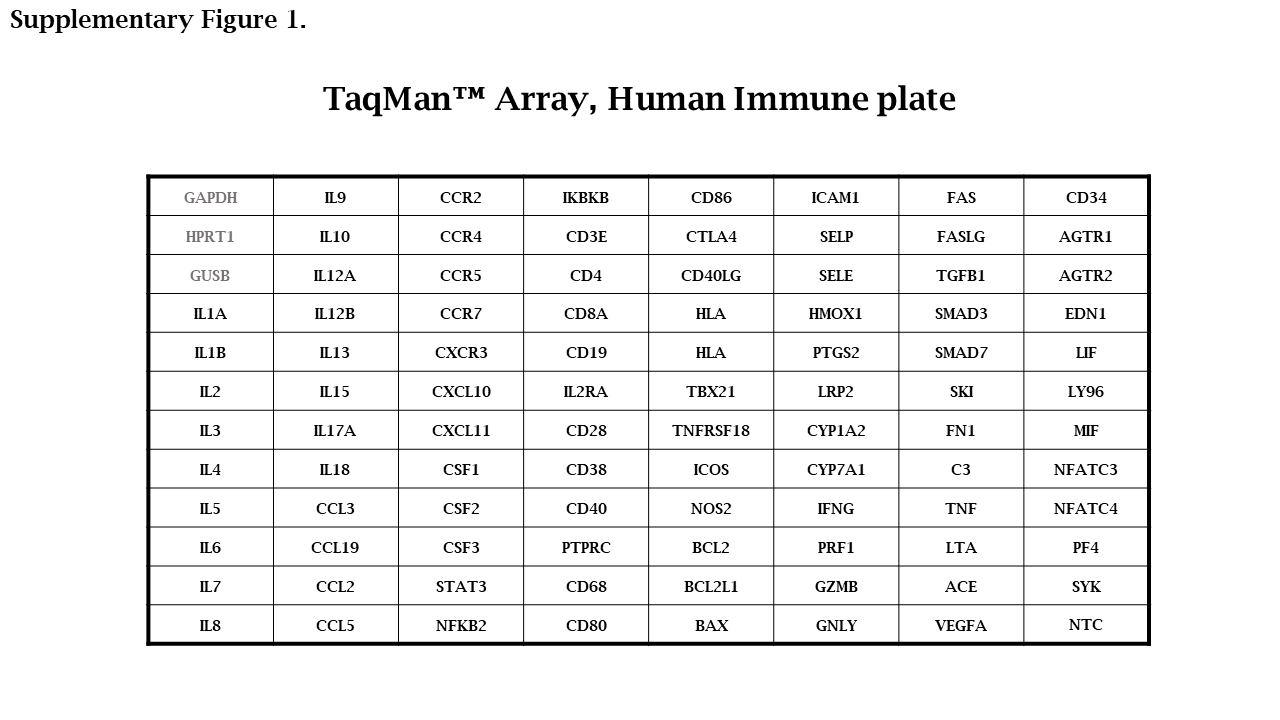

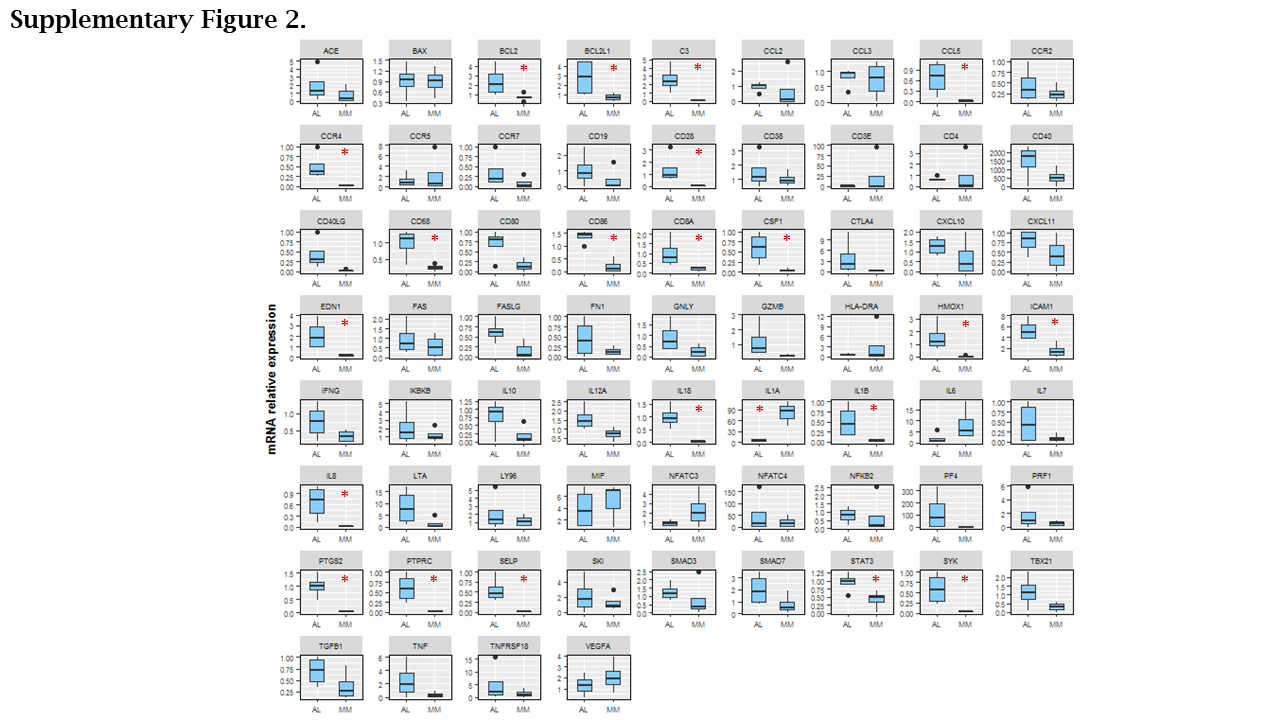


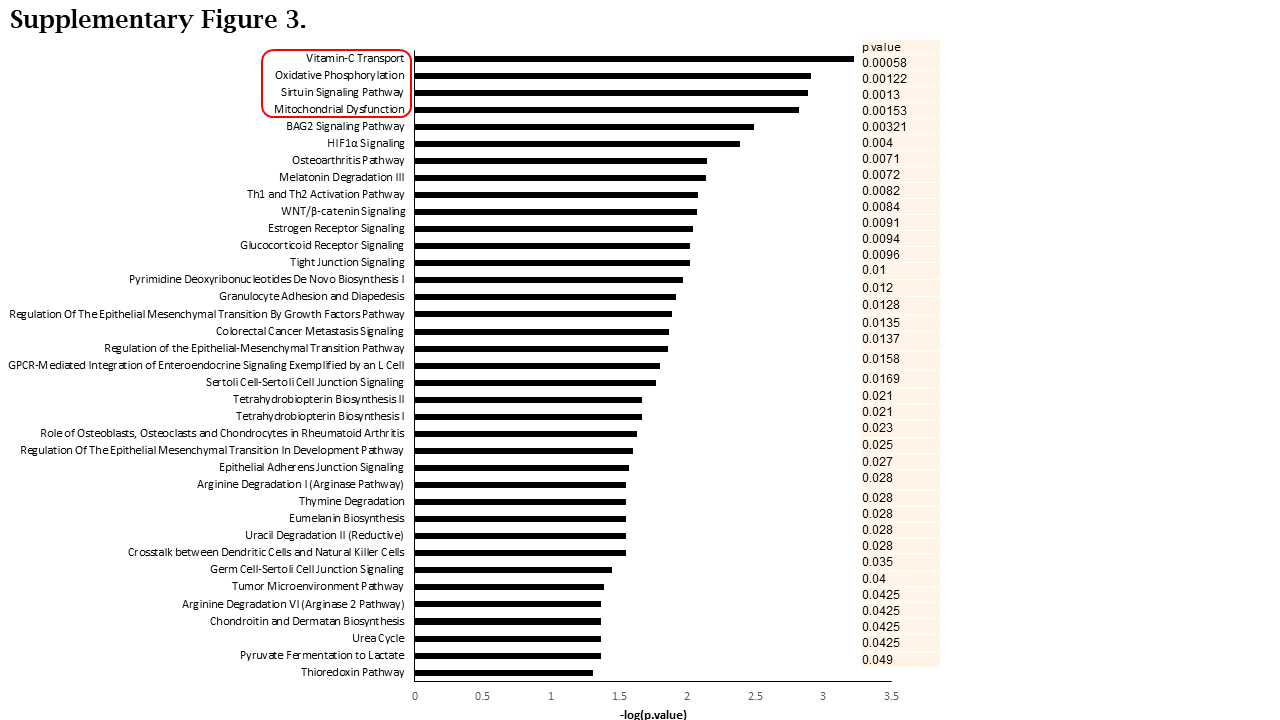

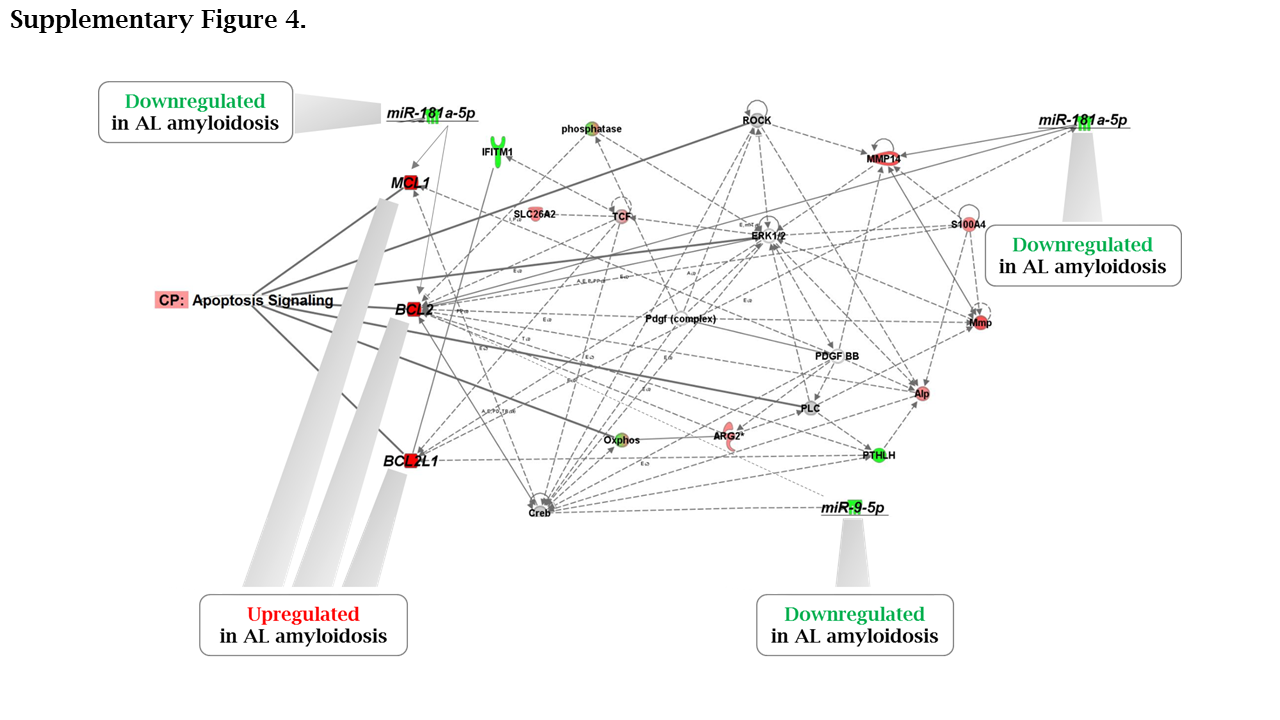

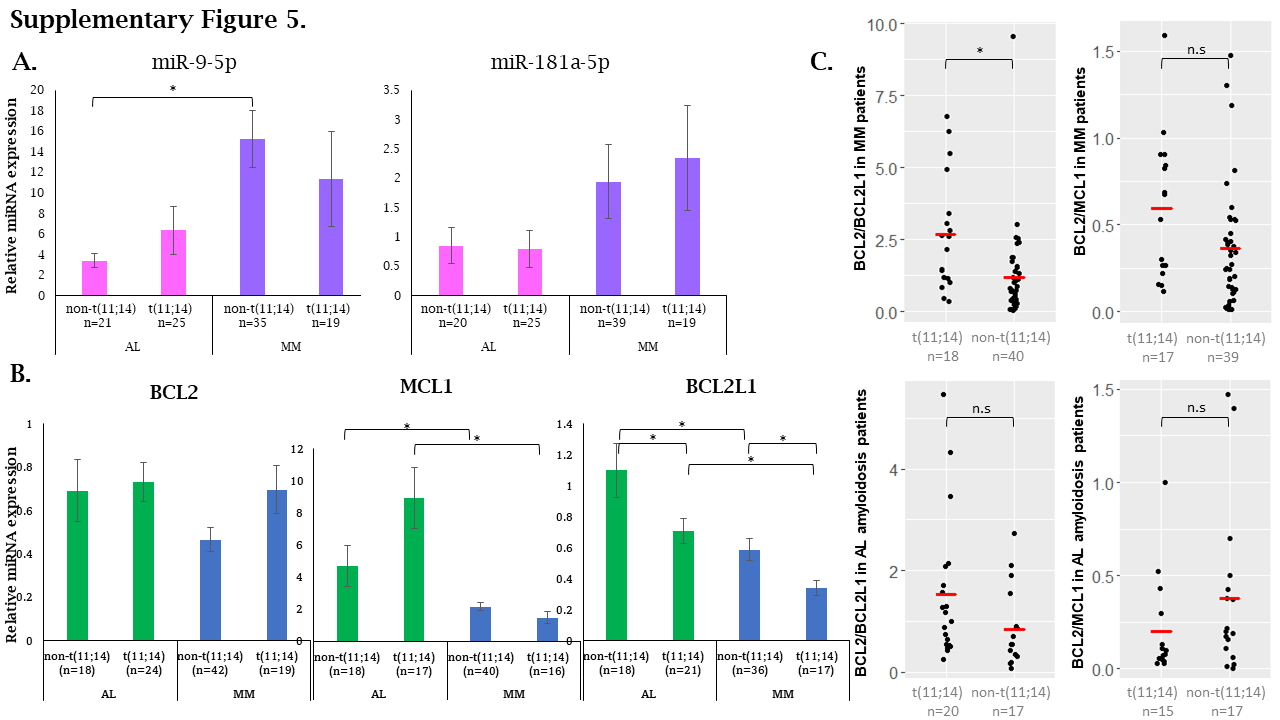

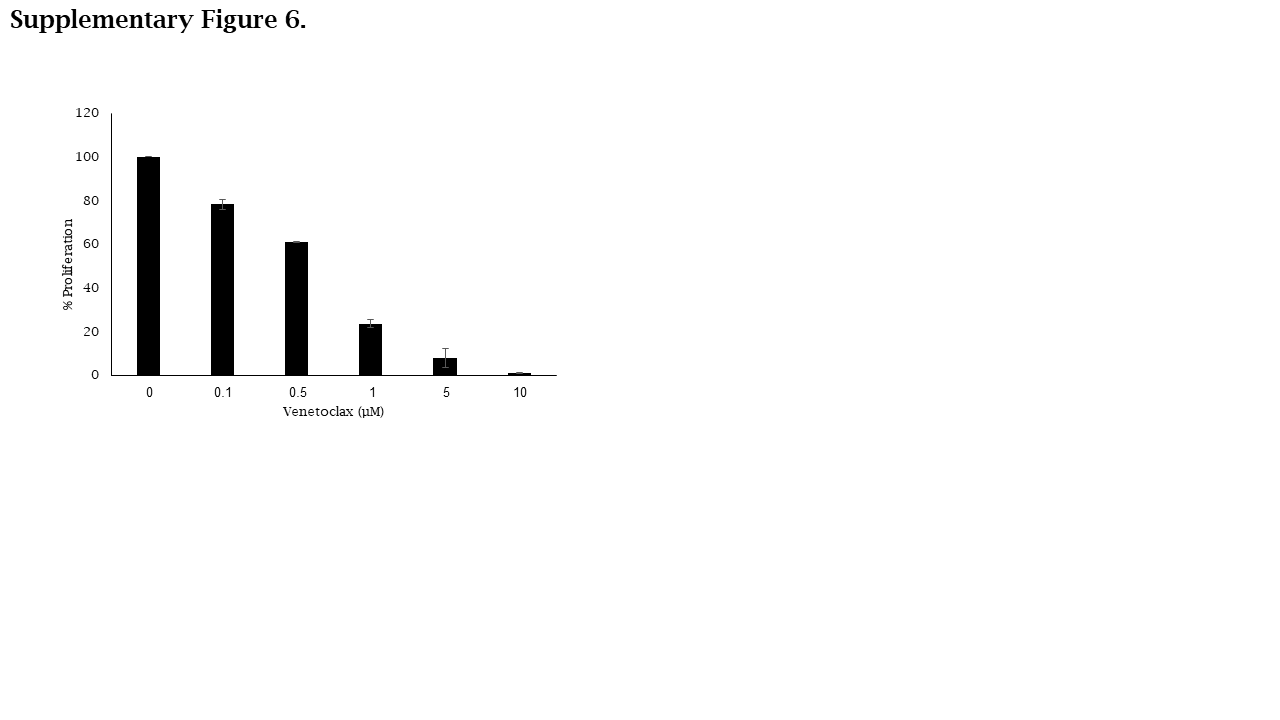


**Supplementary Figures**

**Supplementary Figures**

**Supplementary Figure 1. TaqMan™ Array, human immune plate gene list.** Pre-Amp cDNA samples were loaded on TaqMan™ Array, Human Immune plate coated with probe+primer as indicated in each well.

**Supplementary Figure 2. Gene expression pattern in BM samples of AL amyloidosis (n=4) and MM patients (n=4).** Sixty-seven genes were detected by qRT-PCR, normalized to hypoxanthine phosphoribosyltransferase 1 (HPRT1) and glucuronidase beta (GUSB) endogenous controls. *p<0.05.

**Supplementary Figure 3. Gene enrichment analysis showing mitochondrial signaling pathways as the most related to gene input.** The gene list was downloaded from the GEO database (GSE175384) and reanalyzed. Gene enrichment analysis demonstrated that genes that are related to mitochondrial signaling pathways are aberrantly expressed in AL amyloidosis. The 4 most significantly related pathways to mitochondrial activity are vitamin C transport, oxidative phosphorylation, sirtuin signaling pathway and mitochondria dysfunction.

**Supplementary Figure 4.** **Biological relationship between the downregulated miRNAs and mitochondrial metabolic pathways**. The mitochondrial metabolic pathway (OXPHOS) was found to be a key pathway in AL amyloidosis. The anti-apoptotic genes (MCL1, BCL2 and BCL2L1) were manually added to the analysis in relation to apoptosis signaling. Green and red represent low and high expression, respectively. The straight and dashed lines denote direct and indirect relationships, respectively.

**Supplementary Figure 5. Expression of miR-9-5p, miR-181a-5p and BCL2 family members in AL amyloidosis and MM patients stratified by translocation 11:14.** (A) Analysis of miR-9-5p and miR-181a-5p and (B) BCL2, MCL1 AND BCL2L1 in AL amyloidosis and MM patients by t(11:14). Expression levels were determined by qRT-PCR. (C) BCL2/BCL2L1 ratio and BCL2/MCL1 ratio in MM patients (upper panel) and in AL amyloidosis patients (lower panel). AL, AL amyloidosis; MM, multiple myeloma; qRT-PCR, quantitative real-time polymerase chain reaction.

**Supplementary Figure 6. Treatment with Venetoclax effect cell viability in ALMC-1 cells.** ALMC-1 cells were treated with 0-10µM of venetoclax for 48 hours. Reduced proliferation of cells was measured by WST-1 proliferation assay. The graph denotes the mean + SEM expression from at least 3 experiments.
